# Liverworts of Magadan: Life on the Edge of Beringia

**DOI:** 10.3390/plants12233928

**Published:** 2023-11-22

**Authors:** Vadim A. Bakalin, Ksenia G. Klimova, Daniil A. Bakalin, Seung Se Choi

**Affiliations:** 1 Laboratory of Cryptogamic Biota, Botanical Garden-Institute FEB RAS, Makovskogo Street 142, 690024 Vladivostok, Russia; ksenia.g.klimova@mail.ru (K.G.K.); daniil.bakalin@gmail.com (D.A.B.); 2 Team of National Ecosystem Survey, National Institute of Ecology, Seocheon 33657, Republic of Korea

**Keywords:** North-East Asia, liverworts, hepaticae, beringian land bridge

## Abstract

Magadan Province, located on the southwestern edge of Greater Beringia, combines various floristic elements in its flora, including Beringian and circum-boreal species. The geographic position and habitat diversity of Magadan Province predicts its liverwort flora is one of the richest hemiarctic floras in Asia. The distribution of species throughout the region is uneven, and while its southern part shows close connections with the suboceanic floras of Kamchatka, the middle and central parts are clearly related to the floras of Chukotka, where the Beringian land bridge directly lies in the past. The wide distribution of basic rocks here leads to the existence of a significant complex of basiphylous taxa. A total of 214 taxa (including 205 species and nine varieties) are reported in the present paper. The study of liverworts of Magadan Province should be continued and several new additions to the floral list are highly likely.

## 1. Introduction

Magadan Province is a region in northeast Asia. Most of it is occupied by the Kolyma Plateau, whereas the southern edge is framed by the Sea of Okhotsk. Unlike in the neighboring regions Chukotka, the Republic of Yakutia, Kamchatka Territory and the northern part of Khabarovsk Territory, in this area, with the exception of a narrow strip along the coast, there was almost no indigenous population. This is due to extremely unfavorable living conditions: a small number of wild plants consumed by humans, scarce fish resources, and an ultracontinental climate that dominates most of the region. For a phytogeographer or botanist in a broad sense, Magadan Province is of undoubted interest as a place where tundra–steppe landscapes, similar to those that existed on the Beringian land bridge during the peak of the last glaciation, are preserved over a large area. This is how Magadan Province differs from Chukotka—the central part of the Beringian land bridge in the past. The climate in central Magadan Province has changed little since those times [1,2], while in most of Chukotka, except for the areas adjacent to Magadan Province, it has become much wetter.

The data available in the literature on liverworts of Magadan Province are rather scarce. The first and last checklist of Magadan liverworts was published by Blagodatskikh and Duda [3], including 92 species. This number in itself is not large; moreover, the report describes the distribution of taxa throughout the province quite unsatisfactorily. Historically, even in the 35 years since the publication of the cited checklist, the liverworts of Magadan have remained unstudied except for the efforts of our team. The province does not have “its own” hepaticologist, just as there are none in the neighboring regions, except in Yakutia. Our goal was to conduct a critical assessment of the available literature data, as well as to systematize the material that we collected every year for five years, from 2010 to 2014, in different areas of the region, as well as to analyze the phytogeographical relationships of the studied flora. The results of this effort are presented in the present report.

Thus, before this account, fewer than 100 liverwort species were known from Magadan Province, and their knowledge seemed to be significantly less in comparison with what is known from mosses (364 species) [4] and from vascular plants (1441 species) [5]. This was one of the arguments to conduct the present research.

## 2. Results

### 2.1. General Diversity and Characteristics

A total of 214 taxa were identified, including 205 species and nine varieties. Five species were excluded from the flora. Particularly rare records include *Apotreubia nana* (interpretation follows Bakalin and Vilnet [6]), a species very rare in hemiarctic Beringia and in northeast Asia, known from Magadan locality only. The calciphilous mesophyte *Asterella lindenbergiana*, which has a discontinuous circumpolar range, is also rare in northern Asia. The distribution of *Barbilophozia rubescens* is very poorly studied; in Asian Russia, this is one of the very few localities.

At the northern border of their ranges are *Bazzania denudata* and *B. trilobata*. Their locations in Magadan Province are the northernmost in Asia. For *Moerckia flotoviana*, a suboceanic subcircumpolar boreal–temperate species, this locality is the northernmost in Asia and, apparently, the most continental of those known. The northernmost occurrences of *Crossocalyx hellerianus* and *Scapania apiculata* are also found in the studied flora—these boreal circumpolar epixylous taxa rarely penetrate the Asian hemiarctic. The same should be said of *Neoorthocaculis attenuatus*, although this species was also observed in habitats other than epixylous. A similar situation was observed for *Tritomaria exsecta* and *Tritomaria exsectiformis*. The rare boreal, predominantly suboceanic *Fuscocephaloziopsis loitlesbergeri* is at the northern limit of its range here, and this is one of only a few occurrences of the species in Asia. *Frullania austinii* (=*F. bolanderi* s. auct.), a common epiphyte of boreal Pacific Asia, is found here at the northernmost locality in Asia and in an atypical habitat (the identity of the name may be questioned). The distribution of *Frullania davurica* is also at the northern border here. Very unexpected is a record of *Harpanthus scutatus*, a mainly temperate suboceanic species, found in typical habitats (decaying wood) but in isolation from its range, which mainly ends in Asia at 50 degrees northern latitude. The hemiboreal Asian, mostly suboceanic *Lophozia lantratoviae* is also found here at the northern limit of its distribution. The East Asian temperate *Mylia verrucosa* is known here in isolation from its main range. It should be noted that both these species and the abovementioned taxa of *Bazzania* are found here only in a narrow strip along the coast, where the climate is much milder than in the interior areas. Conventionally, *Metzgeria pubescens* belongs to the same group—a species with a wide range but rarely penetrating the hemiarctic. The East Asian temperate *Radula obtusiloba* was apparently found in relict habitats at the northern border of its distribution, at a significant distance from its area core. Conditionally ‘southern’ species in the flora, in addition to those mentioned, also include all species of *Riccardia*, *Riccia,* and *Ricciocarpos*. In general, for East Siberian *Scapania rufidula*, this is also the northernmost location.

At the southern border of its continuous distribution in continental Asia is *Cryptocolea imbricata*. Although this species even reaches North Sikhote-Alin (48 degrees northern latitude), its locations there are clearly of a relict nature [7]. The same should be said of the distribution of *Eocalypogeia schusteriana*; farther south, the species is found as a relict (for example, in central Sakhalin) [8]. Apparently, the Beringian *Frullania ignatovii* is generally at the southern limit of its distribution in northeast Asia here, although westward, it penetrates farther south to Baikal Siberia [9]. *Frullania subarctica* is very common in the studied area, but its representation in communities is sharply reduced to the south of Magadan Province. *Herbertus arcticus*, a poorly studied species whose taxonomical status may be questioned, is a typical Beringian species, although it is found farther south in several enclaves of the Beringian flora. The same ‘Mega-Beringian’ complex includes *Lejeunea alaskana*, *Lophozia schusteriana* (presumably a species of taxonomically doubtful status), *Marsupella arctica*, *Neoorthocaulis hyperboreus*, *Plagiochila arctica*, *Pseudolepicolea fryei*, *Radula prolifera*, and *Scapania ligulifolia*. Arctic circumpolar *Lophoziopsis rubrigemma* and *Prasanthus suecicus* reflect the Arctic connections of the flora. The southern tip of the Magadan region is the end of the continuous distribution of *Mesoptychia sahlbergii*, although, in enclaves, this species reaches even the Baikal Lake area. The southernmost localities of *Scapania brevicaulis* in Asia are also located here. *Scapania magadanica* deserves special mention—apparently a Beringian species known from areas that could have been nunataks during the Pleistocene–Holocene glaciations [10]. In addition, the species also occupies a special position in the *Scapania* system as a genetically weakly isolated derivate of the strikingly morphologically different *Scapania kaurinii*.

The suboceanic North Pacific *Fuscocephaloziopsis pachycaulis* is found here in the most continental climate conditions known for the species. This partly applies to *Scapania obscura* as well.

The calciphilous complex is quite significant, which is explained by the wide distribution of basic rocks, although they are all of arctic–hemiarctic affinities; there are no southern species among them. Examples include *Arnellia fennica*, *Lophoziopsis pellucida*, *L. polaris*, four species of the genus *Mannia*, seven species of the genus *Mesoptychia* (conventionally including *M. heterocolpos*, although it also grows on substrates other than basic), *Odontoschisma macounii*, *Oleolophozia perssonii*, *Peltolepis quadrata*, *Pseudotritomaria heterophylla*, *Saccobasis* spp., *Sauteria alpina*, and *Scapania gymnostomophila*. Conventionally, *Scapania cuspiduligera*, *Schljakovianthus quadrilobus*, and *Sphenolobus cavifolius* can be included in this group.

### 2.2. List of Taxa

Taxa in the checklist are arranged alphabetically; the nomenclature follows Söderström et al. [11], with the exception of Solenostomataceae, where a narrow genus concept was adopted, acceptance of *Pseudolophozia* Konstant. and Vilnet as distinct from *Barbilophozia* Loeske, the new concept for *Schistochilopsis* (N. Kitag.) Konstant. according to Bakalin et al. [12], and the narrow species concept for *Blepharostoma* (Dumort.) Dumort., following [13]. Each taxon is annotated with (1) reproductive structures (ant.—antheridia; arch.—archegonia; per.—perianths; spor.—sporophytes; gemm.—gemmae), if present; (2) the altitudinal range, in meters above sea level; (3) collection localities (in accordance with that described in the Section 4.2 of the paper), where abbreviations of the floristic districts are given in bold font; (4) a description of the habitat in the area treated; and (5) a brief citation of literature reports, where present.

***Aneura pinguis*** (L.) Dumort.—per., spor., arch.—0–1600—**KOLY** (1, 2, 3), **O-Kol** (5, 6), **OKHO** (8). Wet (with mostly degrading vegetation cover) hollows in oligotrophic and mesotrophic bogs and wet moss tundras; wet crevices in rocks of neutral or alkaline reaction; less often on humus or rocky substrates near watercourses or along the edges of spots of fine soil of cryogenic origin. Hemiarctic forests, crooked forests, tundras, alpine wastelands, coastal rocky slopes.

***Anthelia juratzkana*** (Limpr.) Trevis.—per., ant., spor.—0–1600—**KOLY** (1, 4), **O-Kol** (5, 6), **OKHO** (8). Fine soil spots of cryogenic origin, fine soil, humus and rocky banks of watercourses, wet crevices in rocks, and less often spots of drying peat (in degrading hummocks) in wet tundras. Mainly mountain tundra belt and alpine wastelands, and less often (mainly along the banks of watercourses) a belt of crooked forests or scattered hemiarctic communities with *Larix*. Recorded for **KOLY** and **OKHO** by Blagodatskikh and Duda [3].

**Figure 1:**
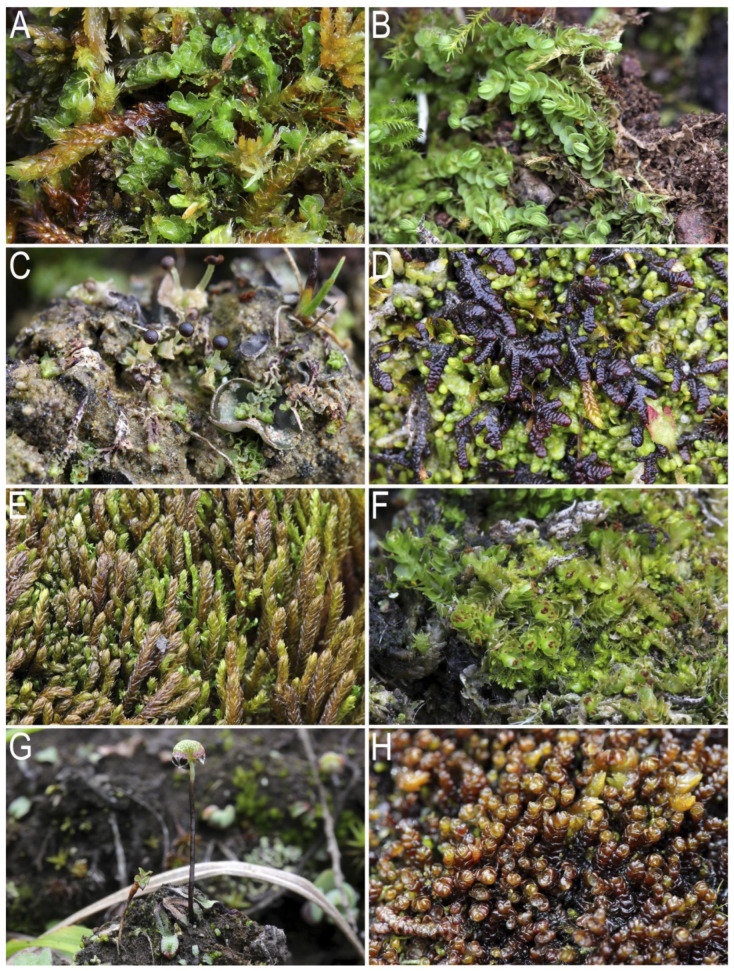
Liverworts in natural conditions in Magadan Province: (**A**) *Apotreubia nana*; (**B**) *Arnellia fennica*; (**C**) *Fossombronia alaskana*; (**D**) *Frullania subarctica*; (**E**) *Herbertus arcticus*; (**F**) *Lophoziopsis polaris*; (**G**) *Mannia gracilis*; (**H**) *Marsupella arctica* (Photo by Choi S.S., 2011).

***Apotreubia nana*** (S.Hatt. et Inoue) S.Hatt. et Mizut.—gemm.—1106—**KOLY** (1). Depressions in an oligotrophic *Sphagnum* swamp, formed in a basin bounded by a moraine(?) ridge. Mountain tundra belt. Figure 1A.

***Arnellia fennica*** (Gottsche and Rabenh.) Lindb.—580–1600—**KOLY** (1, 2), **O-Kol** (6). Wet crevices in limestone or basalt rocks, and less often banks of streams in wet grass-moss communities formed on limestone or along the edges of solifluction spots in basalt-based tundras. Mountain tundra belt. Recorded for **O-Kol** by Blagodatskikh and Duda [3]. Figure 1B.

***Asterella lindenbergiana*** (Corda ex Nees) Arnell—10—**OKHO** (8). Crevices in seacoast cliffs.

***Barbilophozia barbata*** (Schmid. ex Schreb.) Loeske—per. –15–1580—**KOLY** (1, 3, 4), **O-Kol** (5, 6), **OKHO** (7, 8). Moderately moist to dry rocks, boulders, and crevices between them in rocky placers, dry moss-grass slopes in forest and tundra-steppe communities, rarely moderately moist banks of streams (outside the direct influence of flowing water), and hummocks in moss tundras. Coastal rocks, hemiarctic *Larix* forests, crooked forests, and mountain tundras. Recorded for **KOLY** and **OKHO** by Blagodatskikh and Duda [3].

***Barbilophozia hatcheri*** (Evans) Loeske—gemm.—170–1580—**KOLY** (1), **O-Kol** (5, 6), **OKHO** (7, 8). Moderately moistened to dry crevices in rocky fields and gravelly wastelands, fine soil in dwarf shrub-lichen tundras on slopes, less often humus banks of streams, side walls of hummocks in mossy tundras and swamps, and rarely decaying wood. Hemiarctic *Larix* forests, crooked forests, mountain tundra, and alpine wastelands. Recorded for **OKHO** by Blagodatskikh and Duda [3].

***Barbilophozia lycopodioides*** (Wallr.) Loeske—gemm.—350–510—**OKHO** (7, 8). Moderately moistened rock crevices, crevices in a rocky field on slopes. Hemiarctic *Larix* forests and *Pinus pumila* crooked forests. Recorded for **G-Omol** and **OKHO** by Blagodatskikh and Duda [3].

***Barbilophozia rubescens*** (Schust. and Damsh.) Kartt. and L. Söderstr.—gemm.—350—**OKHO** (8). Moderately dry crevices in the rocky field. Crooked forest belt.

***Bazzania denudata*** (Lindenb. et Gottsche) Trevis.—10—**OKHO** (8). Humus soil covering rocks in the *Alnus* forest along the stream. Crooked forest.

***Bazzania trilobata*** (L.) S. Gray—10–350—**OKHO** (8). Near trunk base clumps in sparse *Larix* forest with *Pinus pumila* understory, wet rocks in *Alnus* forest. Crooked forests. Recorded for **OKHO** by Blagodatskikh and Duda [3].

***Biantheridion undulifolium*** (Nees) Konstant. and Vilnet—ant.—1050–1170—**KOLY** (1), **O-Kol** (6). Wet moss (mainly *Sphagnum*) hummocks in tundras (including those developed on limestone) and oligotrophic swamps, and less often moss turf along the edges of fine soil spots of cryogenuic origin. Mountain tundra belt.

***Blasia pusilla*** L.—gemm.—10–1090—**KOLY** (3, 4), **OKHO** (8). Wet shaded loams and sandy loams along riverbanks and roadsides. Hemiarctic *Larix* forests, crooked forests, and mountain tundras. Recorded for **KOLY, O-Kol** and **OKHO** by Blagodatskikh and Duda [3].

***Blepharostoma brevirete*** (Bryhn and Kaal.) Vilnet and Bakalin—per., spor.—510–1600—**KOLY** (1, 2, 4), **O-Kol** (5, 6), **OKHO** (8). The edges of fine soil spots of cryogenic origin in basalt-based tundras, habitats with late-melting snow, less often the side walls of hummocks and humus in degrading hummocky communities developed on rocks of neutral or alkaline composition, fine soil banks of streams, wet crevices in limestone, rarely acidic, and rocks. Crooked forests, mountain tundras, and alpine wastelands.

***Blepharostoma neglectum*** Vilnet & Bakalin—816—**OKHO** (8). Wet mossy mats along lake bank. Crooked forest.

***Blepharostoma trichophyllum*** (L.) Dumort.—per., spor.—15–1400—**KOLY** (1, 2, 3, 4), **O-Kol** (5, 6), **OKHO** (8). Humus and fine soil banks of streams and lakes, side walls of hummocks in mossy tundras and swamps, niches under the roots of *Larix* trees, and crevices in cliffs. Hemiarctic *Larix* forests, crooked forests, and mountain tundras. Recorded for **KOLY, O-Kol,** and **OKHO** by Blagodatskikh and Duda [3].

***Bucegia romanica*** Radian—arch.—1100—**O-Kol** (6). Wet crevices in basalt cliffs near a waterfall. Mountain tundra belt.

***Calycularia laxa*** Lindb. et Arnell—ant., arch., per., spor.—0–1250—**KOLY** (1, 3), **O-Kol** (5), **OKHO** (7, 8). More or less moist, usually moderately to fully shaded rock crevices, as well as moist vertical rocky walls and horizontal rock shelves, rocks and wet humus, and fine soil slopes near watercourses (outside the direct influence of flowing water even during floods); rarely side walls hypnoid moss hummocks in swamps and wet tundras. Hemiarctic *Larix* forests, crooked forests (most often), and mountain tundras. Recorded for **O-Kol** and **OKHO** by Blagodatskikh and Duda [3].

***Calypogeia integristipula*** Steph.—spor., gemm.—0–1000—**KOLY** (3), **O-Kol** (5, 6), **OKHO** (7, 8). Humus slopes to watercourses, moist rocky crevices, niches in slopes covered by hummocky tundra, niches under tree roots, and less often, crevices among stones in rocky fields. Coastal rocks, hemiarctic *Larix* forests, crooked forests, and mountain tundras. Recorded for **KOLY** and **OKHO** by Blagodatskikh and Duda [3].

***Calypogeia muelleriana*** (Schiffn.) Mull.Frib.—spor.—40–1330.—**KOLY** (1, 2, 3, 4), **O-Kol** (5, 6), **OKHO** (8). Humus and fine soil slopes to watercourses and small lakes, decaying wood, side walls of hummocks in oligotrophic swamps, and moist mossy tundras. Hemiarctic *Larix* forests, crooked forests, and mountain tundras. Recorded for **KOLY** and **OKHO** by Blagodatskikh and Duda [3].

***Calypogeia neesiana*** (C. Massal. and Carestia) Müll. Frib.—1090—**KOLY** (4). Drying peat in the moss tundra. Mountain tundra belt. Recorded for **O-Kol** by Blagodatskikh and Duda [3].

***Calypogeia sphagnicola*** (Arnell and J. Perss.) Warnst. and Loeske—gemm.—10–1100—**KOLY** (3, 4), **O-Kol** (5, 6). Moisty peat or *Sphagnum* clumps in moist tundras, moist moss-covered steep slopes, less often along the banks of weakly flowing ponds, and small lakes. Hemiarctic *Larix* forests, mountain tundra belt. Recorded for **KOLY** by Blagodatskikh and Duda [3].

***Cephalozia bicuspidata*** (L.) Dumort.—ant., arch., per.—10–1270—**KOLY** (1, 4), **O-Kol** (5, 6), **OKHO** (7, 8). The side walls of hummocks in moist moss tundras and oligotrophic bogs, wet crevices in rocks and rocky placers, wet roadsides, and the edges of fine soil spots of cryogenic origin. Hemiarctic *Larix* forests, crooked forests, and mountain tundras. Recorded for **KOLY, O-Kol,** and **OKHO** by Blagodatskikh and Duda [3].

***Cephaloziella arctogena*** (Schust.) Konst.—per., ant.—20–900—**KOLY** (4), **O-Kol** (5), **OKHO** (8). Dying *Sphagnum* cushions in moist mossy tundra, side walls of hummocks in dwarf shrub-moss tundra, and more or less dry rocky crevices in the cliffs of acidic composition. Coastal rocks, mountain tundra belt.

***Cephaloziella aspericaulis*** Joerg.—gemm.—880—**OKHO** (7). Wet northeast-facing cliffs. Mountain tundra belt.

***Cephaloziella divaricata*** (Sm.) Schiffn. gemm.—per., ant.—5–1580—**KOLY** (1, 4), **O-Kol** (6), **OKHO** (8). Moist decaying moss turf in mossy tundras, moist crevices in cliffs of neutral or alkaline reaction, fine soil along the edges of fine soil spots of cryogenic origin, humus banks of streams, and moist crevices among stones. Coastal cliffs, hemiarctic forests, crooked forests, mountain tundras, and alpine wastelands.

***Cephaloziella grimsulana*** (Jack ex Gott. and Rabenh.) Lacout—per., ant., arch.—15–1380—**O-Kol** (6), **OKHO** (8). Wet fine soil along the edges of fine soil spots of cryogenic origin in tundras, and rarely moist crevices in coastal rocks. Coastal rocks, mountain tundra belt.

***Cephaloziella hampeana*** (Nees) Schiffn.—15–1400—**KOLY** (1), **O-Kol** (6), **OKHO** (8). Moist rocks along watercourses or near the seashore, edges of fine soil spots of cryogenic origin in mossy tundras. Coastal rocks, crooked forests, and mountain tundra.

***Cephaloziella rubella*** (Nees) Warnst.—ant., arch., per.—300–890—**KOLY** (1, 3), **O-Kol** (5). Decaying wood in *Larix* and *Alnus* thickets, crevices among stones in stony fields, and less often among small pebbles on a steep tundra-steppe covered slope. Hemiarctic *Larix* forests, crooked forests.

***Cephaloziella rubella*** (Nees) Warnst. var. ***bifida*** (Schmid. ex Schreb.) Douin—per., ant., spor.—310—**KOLY** (3). Wet clayey roadside in a swampy *Larix*-Salix dominating forest. Hemiarctic *Larix* forests.

***Cephaloziella spinigera*** (Lindb.) Joerg.—per., ant., arch., spor.—10–1100—**KOLY** (4), **O-Kol** (6), **OKHO** (8). The banks of streams, moist fine soil in spots of cryogenic origin in mossy tundras, and less often the side walls of hummocks in swamps and mossy tundras. Hemiarctic *Larix* forests, crooked forests, and mountain tundras.

***Cephaloziella uncinata*** Schust.—arch., per., gemm.—450–1310—**KOLY** (1), **O-Kol** (6), **OKHO** (8). Moist edges of fine soil spots of cryogenic origin in tundras, moist stones and wet rocks along streams, and hollows in wet tundras. Mountain tundra belt.

***Cephaloziella varians*** (Gottsche) Steph.—per., ant., spor., gemm.—5–1740—**KOLY** (1, 4), **O-Kol** (6), **OKHO** (7, 8). The edges of fine soil spots of cryogenic origin and moist hollows in tundras of various compositions, moderately moist rocky crevices (including those filled with humus, and on the seashore), and humus banks of streams. Coastal cliffs, crooked forests, and mountain tundras. Recorded for **KOLY** by Blagodatskikh and Duda [3] as *Cephaloziella arctica* Bryhn and Douin ex Müll. Frib.

***Chiloscyphus fragilis*** (A.Roth) Schiffn.—1260–1400—**O-Kol** (6). Mossy clumps along streams, a hollow in the moist mossy tundra. Mountain tundra belt.

***Chiloscyphus pallescens*** (Ehrh. ex Hoffm.) Dumort.—1260—**O-Kol** (6). A hollow in moist mossy tundra. Mountain tundra belt.

***Chiloscyphus polyanthos*** (L.) Corda—400—**KOLY** (2). Humus slope to a stream in dense *Alnus* thickets. Crooked forests.

***Cladopodiella fluitans*** (Nees) H. Buch—arch.—0–320—**OKHO** (8). A hollow in the moist mossy tundra, weakly moistened soil among *Pinus pumila* on a slope. Crooked forest belt.

***Clevea hyalina*** (Sommerf.) Lindb.— ant., spor. 1150–1340—**KOLY** (1), **O-Kol** (6). Crumbling fine soil (a product of basalt weathering) along streams and crevices in basalt rocky fields, moderately moist crevices in limestone rocks. Mountain tundra belt and alpine wasteland belt.

***Crossocalyx hellerianus*** (Nees ex Lindenb.) Meyl. Recorded for **O-Kol** by Blagodatskikh and Duda (1988) [3] as *Anastrophyllum hellerianum* (Nees ex Lindenb.) R.M. Schust.

***Cryptocolea imbricata*** R.M. Schust—ant.—1100–1600—**KOLY** (1), **O-Kol** (6). The edge of a fine soil spot of cryogenic origin, moist fine soil along the side of an old road, and a moist moss-covered slope toward a stream. Mountain tundra belt.

***Diplophyllum albicans*** (L.) Dumort.—ant., gemm.—400–1740—**KOLY** (1), **OKHO** (7, 8). Fine soil in the crevices of rocky fields (from acidic to alkaline based rocks), rocks on steep slopes, including along streams (outside the direct influence of flowing water). Crooked forests, mountain tundras. Recorded for **KOLY** and **OKHO** by Blagodatskikh and Duda [3].

***Diplophyllum sibiricum*** Vilnet and Bakalin—per., ant., arch., spor., gemm.—500–1310—**KOLY** (1, 3), **OKHO** (7). Crevices in rocky fields, moist cliffs along streams, and rarely weakly moistened humus on a slope in dwarf shrub-moss tundra. Crooked forests, mountain tundra belts.

***Diplophyllum taxifolium*** (Wahlenb.) Dumort.—ant., per., gemm.—0–1600—**KOLY** (1, 4), **O-Kol** (5, 6), **OKHO** (7, 8). Rocks and their crevices, humus, fine soil and rocky banks of streams, crevices between stones in rocky fields, and rarely rotten wood, humus on steep slopes, on top of *Sphagnum* cushions in dwarf shrub-moss hummocky complexes. Coastal cliffs, hemiarctic *Larix* forests, crooked forests, mountain tundras, and alpine wastelands. Recorded for **KOLY, O-Kol** and **OKHO** by Blagodatskikh and Duda [3].

***Douinia plicata*** (Lindb.) Konstant. et Vilnet—per., gemm.—0–1310—**KOLY** (1), **OKHO** (7, 8). Steep mossy slopes in scattered *Pinus pumila* and *Larix* forests, more or less moist crevices of large-block rocky fields, rarely vegetation cover in mossy tundras, edges of fine soil spots of cryogenic origin, wet cliffs near waterfalls, and humus banks of streams (outside the direct influence of running water even during floods). Hemiarctic *Larix* forests, crooked forests, and mountain tundras. Recorded for **OKHO** by Blagodatskikh and Duda [3] as *Macrodiplophyllum plicatum* (Lindb.) Perss.

***Endogemma caespiticia*** (Lindenb.) Konstant., Vilnet et A. V. Troitsky—ant., arch., per.—10-1250—**KOLY** (1, 3, 4), **OKHO** (8). Moist clay and peaty roadsides and trail sides, fine soil spots of cryogenic origin in tundra. Hemiarctic *Larix* forests, crooked forests, and mountain tundras. Recorded for **KOLY** by Blagodatskikh and Duda [3].

***Eocalypogeia schusteriana*** (S. Hatt. et Mizut.) R.M. Schust.—630–1150—**KOLY** (2), **O-Kol** (6). Wet mossy patches in tundras developed on basalts, and moist crevices in limestone cliffs.

***Eremonotus myriocarpus*** (Carr.) Lindb. and Kaal.—per., ant.—10–880—**OKHO** (7, 8). Moist to moderately dry rocks and their crevices, including cliffs along streams. Crooked forests and mountain tundras.

***Fossombronia alaskana*** Steere and Inoue—per., ant., spor.—1260—**O-Kol** (6). The central part of a clayey fine soil spot of cryogenic origin. Mountain tundra belt. Figure 1C.

***Frullania austinii*** J.J. Atwood, Vilnet, Mamontov, and Konstant.—20—**OKHO** (8)—Crevices among stones in N-facing rocky slope covered with moss-lichen tundra. Crooked forest belt. A very atypical habitat for this predominantly epiphytic taxon. Its identity may be doubted.

***Frullania davurica*** Hampe—1150—**O-Kol** (6). Cleft in basalt rocks. Mountain tundra belt.

***Frullania ignatovii*** Sofronova, Mamontov and Potemkin—1150–1400—**O-Kol** (5, 6). Crevices in basalt(?) cliffs. Mountain tundra belt.

***Frullania subarctica*** Vilnet, Borov., Bakalin—1150–1600—**O-Kol** (5, 6). Moist hollows in mossy tundras, mossy turfs along the banks of streams, and less often moderately dry crevices in cliffs on ridgelines. Mountain tundra. Figure 1D.

***Fuscocephaloziopsis leucantha*** (Spruce) Váňa et L.Söderstr.—10–930—**O-Kol** (5), **OKHO** (8). The upper and side surfaces of hummocks in oligotrophic swamps, peaty banks of streams. Hemiarctic *Larix* forests, crooked forests, and mountain tundras

***Fuscocephaloziopsis loitlesbergeri*** (Schiffn.) Váňa et L.Söderstr.—per., ant.—580– **KOLY** (2). The side walls of mossy hummocks in the humid tundra developed on limestone weathering products. Crooked forests.

***Fuscocephaloziopsis lunulifolia*** (Dumort.) Váňa et L.Söderstr.—0–1056—**KOLY** (1, 3, 4), **O-Kol** (6), **OKHO** (7, 8). Humus-covered slopes to watercourses, bare peat, or the side walls of mossy hummocks in swamps and tundras of various compositions, decaying wood. Hemiarctic forests, crooked forests, and mountain tundra belt. Recorded for **O-Kol** and **OKHO** by Blagodatskikh and Duda [3] as *Cephalozia lunulifolia* (Dumort.) Dumort.

***Fuscocephaloziopsis pachycaulis*** (R.M. Schust.) Váňa and L. Söderstr.—per., ant.—290–900—**KOLY** (4), **OKHO** (7, 8). Crevices in the cliffs near waterfalls, humus slopes toward streams, moss hummocks near ponds and watercourses. Crooked forests and mountain tundras.

***Fuscocephaloziopsis pleniceps*** (Austin) Váňa et L.Söderstr.—per., ant.—660–1600—**KOLY** (3, 4), **O-Kol** (5, 6). Humus and mossy banks of streams, side walls of hummocks in swamps and mossy tundras, fine soil along the edges of fine soil spots of cryogenic origin, and the banks of sluggishly flowing puddles, peaty outcrops on steep slopes. Hemiarctic *Larix* forests, crooked forests, and mountain tundras. Recorded for **KOLY** and **OKHO** by Blagodatskikh and Duda [3] as *Cephalozia pleniceps* (Austin) Lindb.

***Gymnocolea inflata*** (Huds.) Dumort.—per., ant., spor.—50–1260—**KOLY** (1, 3, 4), **O-Kol** (5, 6), **OKHO** (8). Moist hollows in mossy tundras, hollows in oligotrophic bogs, less often moist fine soil along streams and on roadsides, as well as the edges of fine soil spots of cryogenic origin. Hemiarctic *Larix* forests, crooked forests, and mountain tundras. Recorded for **KOLY, O-Kol,** and **OKHO** by Blagodatskikh and Duda [3].

***Gymnomitrion brevissimum*** (Dumort.) Warnst.—ant., arch., per., spor.—450–1120—**KOLY** (1), **OKHO** (8). Crevices among the stones of stony placers, rather dry spots on steep crumbling slopes. Mountain tundra belt. Recorded for **KOLY** by Blagodatskikh and Duda [3] as *Marsupella brevissima* (Dumort.) Grolle.

***Gymnomitrion commutatum*** (Limpr.) Schiffn.—ant., spor.—400–1110—**KOLY** (4), **OKHO** (7, 8). Crevices among stones in rocky fields, patches of bare soil on crumbling slopes, less often wet fine soil along the sides of old roads, and fine soil banks of streams. Crooked forests, mountain tundras.

***Gymnomitrion concinnatum*** (Lightf.) Corda—ant., arch., per., spor.—0–1960—**KOLY** (1, 4), **O-Kol** (5, 6), **OKHO** (7, 8). Moist to moderately dry rocks, including those near streams and waterfalls, crevices between stones in rocky fields, the edges and central parts of fine soil spots of cryogenic origin in the tundra, less often the beds of temporary streams, fine soil and humus near snowfields, rarely roadsides in *Pinus pumila* thickets. Coastal cliffs, crooked forests, mountain tundras, and alpine wastelands. Recorded for **KOLY, O-Kol,** and **OKHO** by Blagodatskikh and Duda [3].

***Gymnomitrion corallioides*** Nees—690–1740—**KOLY** (1, 4), **O-Kol** (6), **OKHO** (7). Moist fine soil and gravel among pebbles in scree and alpine heaths, moderately moist cliff crevices, and edges of fine soil spots of cryogenic origin. Mountain tundra and alpine wasteland. Recorded for **KOLY** by Blagodatskikh and Duda [3].

***Gymnomitrion pacificum*** Grolle—1060—**OKHO** (7). Moderately moist crevices in a rocky field on a steep slope. Mountain tundra belt.

***Harpanthus flotovianus*** (Nees) Nees—Recorded for **OKHO** by Blagodatskikh and Duda [3].

***Harpanthus scutatus*** (F. Weber et D. Mohr) Spruce—400—**KOLY** (2). Decaying wood near a stream in a sparse *Larix* forest. Hemiarctic *Larix* forests.

***Herbertus arcticus*** (Inoue and Steere) Schljakov—1150–1600—**O-Kol** (6). Moderately moist to dry rock crevices and wet moss turf along streams in mossy tundras. Mountain tundras and alpine wasteland. Figure 1E.

***Isopaches bicrenatus*** (Schmid. ex Hoffm.) H. Buch—per., ant., arch., spor., gemm.—310–1554—**KOLY** (1, 2, 4), **OKHO** (8). Moderately dry fine soil on steep tundra-steppe covered slopes and gravelly placers, roadsides in crooked forests, and dry fine soil spots of cryogenic origin in mountain tundras. Crooked forests, mountain tundras, and rarely hemiarctic *Larix* forests. Recorded for **O-Kol** by Blagodatskikh and Duda [3] as *Lophozia bicrenata* (Schmidel ex Hoffm.) Dumort.

***Isopaches decolorans*** (Limpr.) Buch—gemm.—450—**OKHO** (8). Dry spots of bare soil on a steep SW-facing slope. Crooked forests.

***Jungermannia atrovirens*** Dumort.—per., ant.—450–1200—**O-Kol** (6), **OKHO** (8). Wet and submerged stones along the banks of streams. Crooked forests, mountain tundras.

***Jungermannia borealis*** Damsh. and Vana—per.—630–1400—**KOLY** (1, 2), **O-Kol** (6). Moist rocks, including limestone, are usually near streams. Mountain tundra belt.

***Jungermannia exsertifolia*** Steph.—Recorded for **OKHO** by Blagodatskikh and Duda [3].

***Jungermannia eucordifolia*** Schljak.—per., ant.—1100—**O-Kol** (6). Moist bank of a stream in the mossy tundra. Mountain tundra belt. Recorded for **OKHO** by Blagodatskikh and Duda [3] as *J. exsertifolia* ssp. *cordifolia* (Dumort.) Váňa (under variety status).

***Jungermannia polaris*** Lindb.—600–1400—per., ant.—**KOLY** (3), **O-Kol** (5, 6). Rocky crevices filled with fine soil and moist rocks near a stream, edges of fine soil spots of cryogenic origin. Mountain tundra.

***Jungermannia pumila*** With.—per., ant.—400–1400—**O-Kol** (6), **OKHO** (7). Fine soil and humus on slopes to streams, moist rocks. Crooked forests, mountain tundra. Recorded for **OKHO** by Blagodatskikh and Duda [3].

***Lepidozia reptans*** (L.) Dumort.—Recorded for **OKHO** by Blagodatskikh and Duda [3].

***Lejeunea alaskana*** (R.M. Schust. et Steere) H. Inoue et Steere—1050–1580—**O-Kol** (5, 6). Hummocks in moist mossy tundra and among mosses in cushions developed in a depression among the alpine wasteland, wet rocky crevices. Mountain tundras, alpine wasteland.

***Lophocolea heterophylla*** (Schrad.) Dumort.—ant., arch., per., gemm.—300–1100—**KOLY** (3), **O-Kol** (6). Decaying wood in *Alnus* thickets, wet moss-covered slopes to streams. Crooked forest belt. Recorded for **OKHO** by Blagodatskikh and Duda [3].

***Lophocolea minor*** Nees—gemm.—150–840—**KOLY** (2), **O-Kol** (6), **OKHO** (7). Decaying wood in sparse *Larix* forests, and less often rocks along streams. Hemiarctic *Larix* forests, crooked forest belt. Recorded for **O-Kol** and **OKHO** by Blagodatskikh and Duda [3].

***Lophozia fuscovirens*** Bakalin and Vilnet—per., gemm.—5–1150—**O-Kol** (6), **OKHO** (7). Moist cliffs in part shade, bare soil spot of cryogenic origin in mossy tundra, and fine-grained soil in the bed of temporary stream. Crooked forests, mountain tundra. 

***Lophozia guttulata*** (Lindb. et Arnell) A. Evans—per.—5–1180—**O-Kol** (6), **OKHO** (8). Mainly decaying wood and lodging living branches of *Pinus pumila*, and rarely humus banks of streams and small lakes in the tundra belt. Crooked forests, mountain tundras. Recorded for **KOLY** by Blagodatskikh and Duda [3].

***Lophozia heteromorpha*** R.M. Schust. et Damsh.—per., ant.—10–930—**O-Kol** (5), **OKHO** (8). Moist fine soil and rock crevices along a stream. Hemiarctic *Larix* forests.

***Lophozia lantratoviae*** Bakalin—gemm.—890—**O-Kol** (5). Peaty shore of the lake, in the splash zone. Hemiarctic *Larix* forests.

***Lophozia longiflora*** (Nees)—arch., per., gemm.—170–1740—**KOLY** (1, 4), **O-Kol** (5, 6), **OKHO** (7, 8). Crevices among stones in rocky fields, moist crevices in cliffs, side walls of hummocks and depressions in oligotrophic swamps and mossy tundras, edges of fine soil spots of cryogenic origin, humus banks of streams, and less often loamy roadsides in crooked forests. Hemiarctic *Larix* forests (rarely), crooked forests, mountain tundras, and alpine wastelands.

***Lophozia murmanica*** Kaal.—gemm.—1000–1060—**O-Kol** (5, 6). Fine soil in a niche under *Larix* roots, wet fine soil in late snow melting habitats. Crooked forests belt. Recorded for **KOLY** by Blagodatskikh and Duda [3] as *Lophozia groenlandica* Nees.

***Lophozia savicziae*** Schljak.—per., gemm.—290–1740—**KOLY** (1, 4), **O-Kol** (5, 6), **OKHO** (7, 8). The side walls of hummocks and hollows in moss and moss-dwarf shrub tundras, the edges of fine soil spots of cryogenic origin, the banks of streams, between stones in rocky fields, moist rocky crevices (including those along watercourses), and rarely roadsides in *Larix* forests (probably “descended” from the alpine belt). Hemiarctic *Larix* forests (rare), crooked forests, mountain tundras, and alpine wastelands.

***Lophozia schusteriana*** Schljak.—per.—1400–1600—**O-Kol** (5, 6). Rocky crevices near the ridge crest, wet hollows in dwarf shrub-moss tundra. Mountain tundra belt. Recorded for **KOLY** by Blagodatskikh and Duda [3].

***Lophozia silvicola*** H. Buch—per., ant., gemm.—50–1090—**KOLY** (1, 2, 4), **O-Kol** (6), **OKHO** (7, 8). Humus on steep slopes (including slopes to streams) in crooked forests, among mosses in cushions in the ground cover and on decaying wood in sparse *Larix* forests, crevices near streams, and less often depressions and side walls of hummocks in moss and dwarf shrub-moss tundras. Hemiarctic *Larix* forests, crooked forests, and mountain tundras.

***Lophozia silvicoloides*** Kitag.—per., gemm.—450–1110—**KOLY** (1), **O-Kol** (6), **OKHO** (8). Moist stones along streams in crooked forests, among mosses in hummocks in the ground cover of *Larix* forests. Hemiarctic *Larix* forests, crooked forests. Recorded for **KOLY** by Blagodatskikh and Duda [3].

***Lophozia ventricosa*** (Dicks.) Dumort.—per., ant., spor., gemm.—10–1300—**KOLY** (1, 3, 4), **O-Kol** (5, 6), **OKHO** (7, 8). Crevices between stones in rocky fields, humus banks of watercourses and small lakes, moist moss clumps and side walls of hummocks, on slopes and flat surfaces in forests and tundras, rocky crevices and niches, including those near watercourses, and rarely patches of bare soil near snowfields. Hemiarctic *Larix* forests, crooked forests, and mountain tundras. Recorded for **KOLY, O-Kol,** and **OKHO** by Blagodatskikh and Duda [3].

***Lophozia wenzelii*** (Nees) Steph. var. ***lapponica*** H. Buch et S.W. Arnell—1400—**O-Kol** (6). Moist cliffs near streams. Crooked forest. This specimen (Mag-51-10-11) contains the form with brownish gemmae and its taxonomic position remains unclear.

***Lophozia wenzelii*** (Nees) Steph. var. ***wenzelii*** gemm.—per.—780—**KOLY** (1). Over *Sphagnum* cushions in hollows in a ridge-hollow swamp along the lake shore. Crooked forest. Recorded for **KOLY** and **OKHO** by Blagodatskikh and Duda [3].

***Lophoziopsis excisa*** (Dicks.) Konstant. et Vilnet var. *excisa*—per., ant., spor., gemm.—310–1310—**KOLY** (1, 2, 4), **O-Kol** (6), **OKHO** (7). Moss-covered steep slopes in *Larix* forests (where on bare ground or among mosses and other liverworts), moderately moist to moderately dry crevices in rocky fields, among mosses in moss hummocks in the tundra, and rarely moist loamy roadsides. Hemiarctic *Larix* forests, mountain tundras. Recorded for **KOLY** by Blagodatskikh and Duda [3] as *Lophozia excisa* (Dicks.) Dumort.

***Lophoziopsis excisa*** var. ***elegans*** (R.M.Schust.) Konstant. et Vilnet—1050—**O-Kol** (6). The edge of a fine soil spot of cryogenic origin in the tundra. Mountain tundra.

***Lophoziopsis longidens*** (Lindb.) Konstant. et Vilnet—per., arch., gemm.—100–1600—**KOLY** (1, 4), **O-Kol** (5, 6), **OKHO** (7, 8). Crevices among stones in rocky field, moderately moist mossy hummocks in the tundra, decaying wood in *Larix* forests and *Pinus pumila* crooked forests, less often moist rocks along streams, humificated soil on steep slopes to watercourses (outside the direct influence of flowing water), and over protruding *Larix* roots. Hemiarctic *Larix* forests, crooked forests, and mountain tundras. Recorded for **KOLY** and **OKHO** by Blagodatskikh and Duda [3] as *Lophozia longidens* (Lindb.) Macoun.

***Lophoziopsis pellucida*** (R.M.Schust.) Konstant. et Vilnet var. *pellucida*—arch., gemm.—580–1600—**KOLY** (1, 2), **O-Kol** (5, 6). Rocks, stones, and fine soil along streams in the areas of alkaline bedrock distribution, moist basalt rocks, and less often moist hollows and edges of fine soil spots of cryogenic origin in mossy and dwarf shrub tundras developed on products of the weathering of limestone and basalts. Mountain tundra belt.

***Lophoziopsis pellucida*** var. ***minor*** (R.M.Schust.) L.Söderstr. et Váňa—gemm.—1180–1400—**O-Kol** (5, 6). Moist basalt rocks along the stream and moist hollows in the dwarf shrub-lichen tundra. Mountain tundra belt.

***Lophoziopsis polaris*** (R.M.Schust.) Konstant. et Vilnet var. *polaris*—per., gemm.– 400–1600—**KOLY** (1, 2, 4), **O-Kol** (5, 6). Wet hollows in mossy tundras, less often *Larix* forests developed over weathering products of limestones or basalts, humus banks of streams, side walls of hummocks in hummocky complexes along the lake shores, moist crevices between stones in stony fields, and rarely in niches under the roots of *Larix* trees on a steep moist slope with basalt rocky outcrops. Hemiarctic *Larix* forests, crooked forests, and mountain tundras. Figure 1F.

***Lophoziopsis polaris*** var. ***sphagnorum*** (R.M.Schust.) Konstant. et Vilnet—gemm.—400–1200—**KOLY** (2, 4), **O-Kol** (5, 6). Hollows in the *Sphagnum* tundra, hollows in ridge-hollow dwarf shrub-moss community along the shore of a lake, humus slope to a stream in an *Alnus* forest, moist humus along streams, and moist crevices between stones in rocky fields. Crooked forests, mountain tundras.

***Lophoziopsis propagulifera*** (Gottsche) Konstant. et Vilnet—ant., arch., per., gemm.—1580—**O-Kol** (6). The edge of a fine soil spot of cryogenic origin in the tundra. Mountain tundra belt.

***Lophoziopsis rubrigemma*** (R.M.Schust.) Konstant. et Vilnet—1150—**O-Kol** (6). A crevice between stones in rocky fields. Mountain tundra belt.

***Mannia gracilis*** (F. Weber) D.B. Schill and D.G. Long—ant., arch., spor.—970-1150– **O-Kol** (6). Moist fine soil on crumbling slopes to streams, cliff crevices near streams. Mountain tundra belt. Figure 1G.

***Mannia pilosa*** (Horn) Frey et Clark—spor.—970–1150—**O-Kol** (6). Crevices between stones in rocky fields, crumbling fine soil (products of basalt weathering) near streams. Mountain tundra belt.

***Mannia sibirica*** (Mull.Frib.) Frey et Clark—1100—**O-Kol** (6). Moist fine soil (basalt weathering products) slope to the stream. Mountain tundra belt.

***Mannia triandra*** (Scop.) Grolle.—spor.—1150—**O-Kol** (6). Crevices among the stones in rocky fields. Mountain tundra. Originally recorded from this locality by Borovichev and Bakalin [14].

***Marchantia alpestris*** (Nees) Burgeff—gemm.—330–1270—**KOLY** (4), **O-Kol** (6). Moist sandy and humus banks of streams in crooked forests, and less often crevices among stones in rocky fields. Crooked forest, mountain tundra belts.

***Marchantia latifolia*** Gray—arch., gemm—5–1120—**KOLY** (1, 2, 3), **O-Kol** (6), **OKHO** (8). Peaty banks of streams, grass-moss communities over limestone weathering products, and less often moist rocks along watercourses. Hemiarctic *Larix* forests, crooked forests. Recorded for **KOLY, O-Kol** and **OKHO** by Blagodatskikh and Duda [3] as *M. polymorpha*.

***Marchantia polymorpha*** L. (=*M. aquatica* (Nees) Burgeff)—gemm.—15—**OKHO** (8). Stones in the dry riverbed of a small oxbow. Hemiarctic *Larix* forest.

***Marsupella apiculata*** Schiffn.—ant., arch., spor.—400–1960—**KOLY** (1, 4), **O-Kol** (6), **OKHO** (7, 8). Fine soil in rock crevices and among stones in rocky fields and gravelly screes, moist loams near snowfields, moist fine earth on fine soil spots of cryogenic origin, and on steep slopes in the tundra. Crooked forests (rarely), mountain tundras, and alpine wastelands. Recorded for **KOLY** by Blagodatskikh and Duda [3] as *Gymnomitrion apiculatum* (Schiffn.) Müll. Frib.

***Marsupella arctica*** (Berggr.) Bryhn et Kaal.—1166—**O-Kol** (6)—The bed of a sluggishly flowing stream in dwarf shrub-moss tundra. Mountain tundra belt. Figure 1H.

***Marsupella boeckii*** (Aust.) Lindb. ex Kaal.—per., ant., spor.—0–1600—**KOLY** (1, 4), **OKHO** (7, 8). Moist rocks and stones near streams and waterfalls, rarely moist edges of fine soil spots of cryogenic origin, and dense fine soil near streams in mountain tundras. Mountain tundras, and less often hemiarctic *Larix* forests and crooked forests. Recorded for **OKHO** by Blagodatskikh and Duda [3].

***Marsupella condensata*** (Aongstr. ex C. Hartm.) Lindb. ex Kaal.—ant., per.—290–1600—**KOLY** (4), **OKHO** (8). Dense fine soil near streams, and fine soil spots in the tundra. Crooked forests, mountain tundra belt.

***Marsupella emarginata*** (Ehrh.) Dumort.—ant., per., spor.–0–1740—**KOLY** (1, 4), **O-Kol** (6), **OKHO** (7, 8). Moist rock crevices, including those near watercourses, rocks or fine soil on banks of streams, wet rocks near waterfalls, and less commonly, wet crevices between stones in rock field. Coastal cliffs, crooked forests, and mountain tundras. Recorded for **KOLY** and **OKHO** by Blagodatskikh and Duda [3].

***Marsupella sprucei*** (Limpr.) H.Bern.—ant., arch., per., spor.—15–1960—**KOLY** (1, 4), **O-Kol** (6), **OKHO** (7, 8). Fine soil in cliff crevices and crevices between boulders in rock field, edges and central parts of moist fine soil spots of cryogenic origin, and less often moist rocks near streams. Coastal cliffs, mountain tundra.

***Mesoptychia badensis*** (Gottsche ex Rabenh.) L.Söderstr. et Váňa—200–1340—**KOLY** (1, 3), **O-Kol** (6). Moist limestone and basalt rocks, including those near streams, and less often basaltic fine soil on the slope to streams. Mountain tundra belt, and less often crooked forests.

***Mesoptychia bantriensis*** (Hook.) L.Söderstr. et Váňa—ant., arch., per.—880–900—**O-Kol** (5). Depressions in ridge-hollow complexes along lake shores (on rocks with a high lime content). Mountain tundra belt.

***Mesoptychia collaris*** (Nees) L.Söderstr. et Váňa—ant., arch., per.—630–1400—**KOLY** (1, 2), **O-Kol** (6). Moist crevices in limestone rocks, moist humus on slopes to streams and crevices in basalt rock fields. Mountain tundra belt. Recorded for **KOLY** by Blagodatskikh and Duda [3] as *Lophozia collaris* (Nees) Dumort.

***Mesoptychia gillmanii*** (Austin) L.Söderstr. et Váňa—ant., arch., per., spor.—400–1400—**KOLY** (1, 2), **O-Kol** (5, 6). Peaty and fine soil banks of streams in tundras developed on weathering products of basalts and limestones, moist crevices between stones in rock fields composed of basalts, moist basalt cliffs near waterfalls, and less often moist depressions in mossy tundras. Crooked forest, mountain tundra belts.

**Figure 2:**
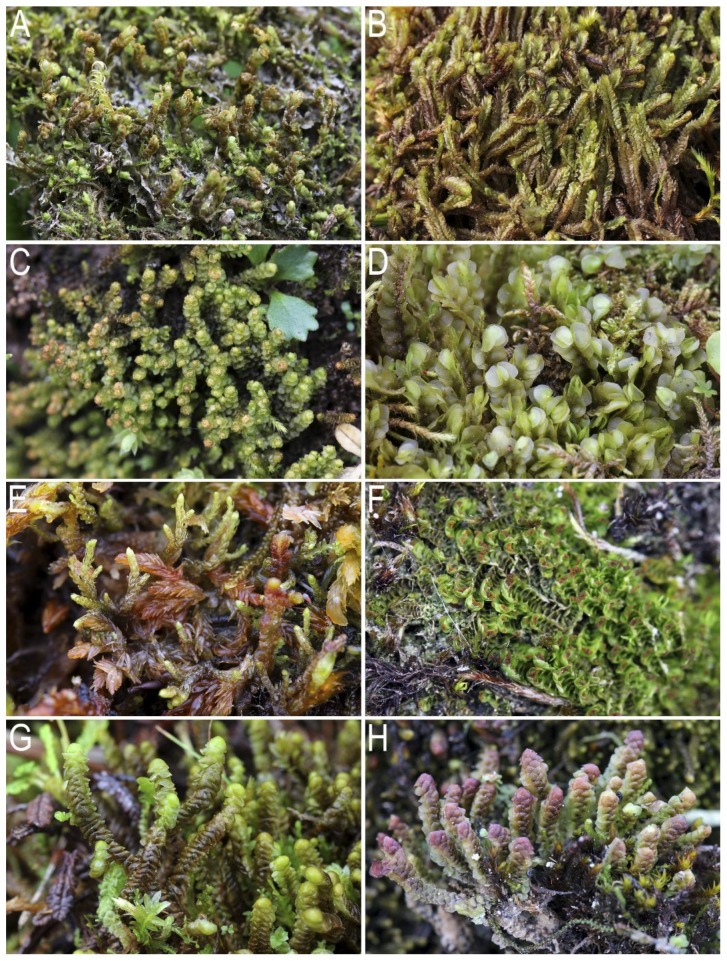
Liverworts in natural conditions in Magadan Province: (**A**) *Mesoptychia heterocolpos*; (**B**) *Mesoptychia sahlbergii*; (**C**) *Neoorthocaulis floerkei*; (**D**) *Plagiochila arctica*; (**E**) *Pseudolepicolea fryei*; (**F**) *Scapania gymnostomophila*; (**G**) *Scapania simmonsii*; (**H**) *Scapania spitsbergensis* (Photo by Choi S.S., 2011).

***Mesoptychia heterocolpos*** (Thed. ex Hartm.) L.Söderstr. et Váňa var. *heterocolpos*—per., gemm.—630–1600—**KOLY** (1, 2), **O-Kol** (5, 6), **OKHO** (7). Peaty steep slopes to streams (usually outside the zone of influence of water, even during floods), crevices between stones in rock fields, moist shaded crevices in limestone and basalt rocks, depressions in tundras of various compositions, less often the edges of fine soil spots of cryogenic origin in various tundras, and rarely decaying wood in hemiarctic *Larix* forests. Hemiarctic *Larix* forests, crooked forests, and mountain tundras. Recorded for **KOLY** by Blagodatskikh and Duda [3] as *Lophozia heterocolpos* (Thed. ex Hartm.) M. Howe. Figure 2A.

***Mesoptychia heterocolpos*** var. ***arctica*** (S.W.Arnell) L.Söderstr. et Váňa—1600—**O-Kol** (6). Moist bank of a stream. Mountain tundra belt.

***Mesoptychia rutheana*** (Limpr.) L.Söderstr. et Váňa—per., ant., arch.—580–1600—**KOLY** (1), **O-Kol** (6). Moist mossy tundras developed on weathering products of limestones and basalts, and less often peaty banks of streams. Mountain tundra belt.

***Mesoptychia sahlbergii*** (Lindb.) A.W. Evans—190–1600—**KOLY** (1), **O-Kol** (5, 6). Moist moss-covered N-facing rocks, steep slopes to streams in mossy tundras, less often hollows in moss tundras and the edges of fine soil spots of cryogenic origin, steep moss-covered slopes in crooked forests and sparse *Larix* forests, and hummocky complexes along the lake shores. In areas of distribution of bedrocks. Mountain tundra belt, and less often crooked forests and hemiarctic *Larix* forests. Recorded for **KOLY** by Blagodatskikh and Duda [3]. Figure 2B.

***Metzgeria pubescens*** (Schrank) Kuwah.—1150—**O-Kol** (6). A fairly dry cleft in the S-facing cliff. Mountain tundra belt. Recorded for **OKHO** by Blagodatskikh and Duda [3].

***Moerckia flotoviana*** (Nees) Schiffn.—ant., per.—1050—**O-Kol** (6). Peaty bank of a stream in wet mossy tundra on the products of basalt weathering. Mountain tundra belt. Recorded for **OKHO** by Blagodatskikh and Duda [3] as *Moerckia hibernica* (Hook.) tsche.

***Mylia anomala*** (Hook.) S.Gray—gemm.—370–1270—**KOLY** (1, 3, 4), **O-Kol** (5, 6). Apices and side walls of hummocks in moist mossy tundras (including those on steep slopes) and on oligotrophic bogs, and less often in communities with Betula middendorfii dominance, on mossy banks of sluggishly flowing streams. Crooked forest and mountain tundra belts. Recorded for **KOLY, O-Kol** and **OKHO** by Blagodatskikh and Duda [3].

***Mylia taylorii*** (Hook.) S. Gray—1270—**KOLY** (4). Over moist *Sphagnum* hummocks. Mountain tundra belt.

***Mylia verrucosa*** Lindb.—per.—10—**OKHO** (8). Humus soil covering rocks along a temporary stream in moist *Alnus* forest on a slope. Crooked forest belt.

***Nardia breidleri*** (Limpr.) Lindb.—510–700—**OKHO** (7). Moist humus soil on a slope in the dwarf shrub-moss tundra, fine soil spots of cryogenic origin. Mountain tundra belt, and rarely crooked forests.

***Nardia geoscyphus*** (De Not.) Lindb.—ant., arch., per., spor.—10–1400—**KOLY** (1, 4), **O-Kol** (5, 6), **OKHO** (7, 8). Moist fine soil spots of cryogenic origin in the tundras, moist soil, rocks and stones along streams, fine soil in snowbed habitats, less commonly moist rock crevices (usually filled with fine soil), peaty soil on mammal paths and clayey roadsides. Hemiarctic *Larix* forests, crooked forests, and mountain tundras. Recorded for **KOLY** and **OKHO** by Blagodatskikh and Duda [3].

***Nardia insecta*** Lindb. per., ant., spor.—650–1300—**KOLY** (1, 4), **O-Kol** (6). The edges of fine soil spots of cryogenic origin, fine soil in the beds of temporary watercourses, and clay soil along the banks of streams. Mountain tundra belt.

***Nardia japonica*** Steph.—ant., arch., per., spor.—10–1250—**KOLY** (1, 4), **OKHO** (8). Banks of streams, peaty soil on the roadsides, trails, peaty side walls of hummocks, and patches of fine soil in tundras. Hemiarctic *Larix* forests, crooked forests, and mountain tundras. 

***Nardia scalaris*** S.Gray—880—**OKHO** (7). Moist crevices in NW-facing rocks. Mountain tundras. Recorded for **KOLY** and **OKHO** by Blagodatskikh and Duda [3].

***Neoorthocaulis attenuatus*** (Mart.) (Mart.) L.Söderstr., De Roo et Hedd.—ant., gemm.—100–1310—**KOLY** (1), **OKHO** (7, 8). Shaded crevices in a large-block rock field, thick living branches of *Pinus pumila* lying on the soil. Crooked forests and rarely mountain tundras.

***Neoorthocaulis binsteadii*** (Kaal.) L.Söderstr., De Roo et Hedd.—ant., arch., per., gemm.—10–1580—**KOLY** (1, 2, 3, 4), **O-Kol** (5, 6), **OKHO** (8). A moss-covered bank of a sluggishly flowing pond, hollows and the upper surface of hummocks in moist mossy tundras and oligotrophic bogs, peaty slopes to a stream, side walls of hummocks on moist steep slopes, and less often moss turf along the edges of fine soil spots of cryogenic origin and moist cliff crevices along streams. Hemiarctic *Larix* forests, crooked forests, mountain tundras, and rarely alpine wastelands. Recorded for **KOLY** by Blagodatskikh and Duda [3] as *Lophozia binsteadii* (Kaal.) A. Evans.

***Neoorthocaulis floerkei*** (F.Weber et D.Mohr) L.Söderstr., De Roo et Hedd.– 650–1050—**KOLY** (1), **O-Kol** (5). Moist hollows in the mountain tundra, moist moss-covered slopes in the tundra. Mountain tundra belt. Figure 2C.

***Neoorthocaulis hyperboreus*** (Schust.) L.Söderstr., De Roo et Hedd.—per.—580–1180—**KOLY** (2), **O-Kol** (6). Moist hollows between hommocks in mossy tundras developed over the weathering products of limestones and basalts.

***Obtusifolium obtusum*** (Lindb.) S.W. Arnell—800—**KOLY** (4). Moist moss patches near a stream. Mountain tundra belt.

***Odontoschisma elongatum*** (Lindb.) Evans—300–890—**O-Kol** (5), **OKHO** (8). Peaty soil along streams in oligotrophic swamps, hollows in hummocky communities along the shores of lakes, and rarely decaying wood. Hemiarctic *Larix* forests belt.

***Odontoschisma macounii*** (Aust.) Underw.– 580–1400—**KOLY** (1, 2), **O-Kol** (5, 6). The side walls and upper surface of hummocks in moist mossy tundras developed on the products of the destruction of limestones and basalts, peat outcrops along the lake shores, the edges of fine soil spots of cryogenic origin, and crevices in large-block rocky fields. Hemiarctic *Larix* forests, mountain tundra belts.

***Oleolophozia perssonii*** (H.Buch et S.W.Arnell) L.Söderstr., De Roo et Hedd.—gemm.—510–1460—**KOLY** (1, 3). Fine soil on the roadside in the area of an old limestone open pit, moist fine soil on a steep slope in the area of limestone distribution. Crooked forests, mountain tundra belts.

***Pellia neesiana*** (Gott.) Limpr.—arch., per.—10–920—**KOLY** (4), **O-Kol** (5), **OKHO** (7, 8). Wet fine soil along streams, peaty lake shores, moist shaded humus slopes, and less often moist clayey roadsides. Hemiarctic *Larix* forests, crooked forests, and less often mountain tundras. Recorded for **OKHO** by Blagodatskikh and Duda [3].

***Peltolepis quadrata*** (Saut.) Mull.Frib.—arch., spor.—690–1600—**O-Kol** (5, 6), **OKHO** (7). Crumbling basalt fine soil to streams, humus-rich banks of watercourses, moist crevices in cliffs. Crooked forests, mountain tundras.

***Plagiochila arctica*** Bryhn et Kaal.—500–1600—**KOLY** (1, 3, 4), **O-Kol** (5, 6). Hummocks and shallow hollows in moist mossy tundras (on flat surfaces and slopes), moss cushions along the edges of fine soil spots of cryogenic origin, and less often crevices between stones in rock fields and moist cliff crevices. As a rule, in places where alkaline rocks occur (basalts, serpentinites, limestones). Mountain tundra belt, alpine wastelands. Figure 2D.

***Plagiochila porelloides*** (Torrey ex Nees) Lindenb. var. ***subarctica*** (Joerg.) Lammes—1180—**O-Kol** (6). Wet soil along the edge of a fine soil spot of cryogenic origin. Mountain tundra belt. Recorded for **O-Kol** and **OKHO** by Blagodatskikh and Duda [3].

***Pleurocladula albescens*** (Hook.) Grolle—per., spor.—10–1600—**KOLY** (1, 4), **O-Kol** (5), **OKHO** (7, 8). Rocks, humus, moss cushions, and fine soil near waterfalls and streams (rarely submerged in temporary watercourses), edges of fine soil spots of cryogenic origin, and rarely moist loamy roadsides, side walls of hummocks in moss tundras. Coastal cliffs, crooked forests, mountains, and inverse tundras. Recorded for **KOLY, O-Kol,** and **OKHO** by Blagodatskikh and Duda [3].

***Prasanthus suecicus*** (Gott.) Lindb.—1550—**KOLY** (1). Spots of fairly dry fine soil of cryogenic origin on a slope in the *Dryas* tundra.

***Preissia quadrata*** (Scop.) Nees—arch., ant., spor.—5–1600—**KOLY** (1, 2, 3, 4), **O-Kol** (5, 6), **OKHO** (7, 8). Peaty banks of streams, crevices between stones in rock fields, cliff crevices (including those near watercourses), edges and central parts of fine soil spots of cryogenic origin, and rarely loamy roadsides. Coastal rocks, hemiarctic *Larix* forests, crooked forests, and mountain tundras. Recorded for **OKHO** by Blagodatskikh and Duda [3].

***Protochilopsis grandiretis*** (Lindb. ex Kaal.) A.V. Troitsky, Bakalin and Fedosov—per., spor., gemm.—370–1400—**KOLY** (1, 2, 3, 4), **O-Kol** (5, 6). Peaty and *Sphagnum*-covered slopes to streams, peat outcrops in hollows between hummocks in oligotrophic bogs, edges of moist fine soil spots of cryogenic origin, and side walls of hummocks in moist mossy tundras. Crooked forests, mountain tundra belt.

***Pseudolepicolea fryei*** (Perss.) Grolle et Ando—per.—1050–1110—**KOLY** (1), **O-Kol** (5, 6). A moss-covered bank of a sluggishly flowing watercourse in the tundra, hollows between hummocks in *Sphagnum* tundra, dying *Sphagnum* hummocks near a temporary watercourse. Mountain tundra belt. Figure 2E.

***Pseudolophozia debiliformis*** (R. M. Schust.) Konstant. and Vilnet—ant., gemm.—10-1600—**KOLY** (1, 4), **OKHO** (7, 8). Crevices between stones in rock fields, crevices and vertical surfaces of cliffs, and edges of fine soil spots of cryogenic origin. Coastal rocks, hemiarctic *Larix* forests, crooked forests, mountain tundra belt.

***Pseudolophozia sudetica*** (Nees ex Huebener) Konstant. and Vilnet var. *sudetica*—per., ant., gemm.—0–1560—**KOLY** (1, 4), **O-Kol** (5, 6), **OKHO** (7, 8). Crevices between stones in rocky fields, cliff crevices, including those near watercourses and waterfalls, stones (often sometimes submerged in water), fine soil and humus along streams, patches of bare soil near snowfields, fine soil along roadsides, edges of fine soil spots of cryogenic origin, and rarely on drying out areas of hummocks in hummocky tundras. Hemiarctic *Larix* forests, crooked forests, and mountain tundras. Recorded for **KOLY** and **OKHO** by Blagodatskikh and Duda [3] as *Lophozia sudetica* (Nees ex Huebener) Grolle.

***Pseudolophozia sudetica*** var. ***anomala*** (Schljakov) Konstant. and Vilnet—gemm.—660—**KOLY** (1). Fine soil along the edge of the snowfield. Mountain tundra.

***Pseudotritomaria heterophylla*** (R.M. Schust.) Konstant. and Vilnet—arch., ant., per., spor., gemm.—970–1600—**KOLY** (1), **O-Kol** (5, 6). Fine soil between hummocks in moss tundras, edges of fine soil spots of cryogenic origin, fine soil with a high lime content on the slope, and less often crevices in basalt rocks. In places where basalts and limestones occur. Mountain tundra, and rarely crooked forest belt.

***Ptilidium ciliare*** (L.) Hampe—10–1600—**KOLY** (1, 2, 3, 4), **O-Kol** (5, 6), **OKHO** (7, 8). In the vegetation cover of tundras of various composition, the edges of solifluction spots, steep moss-covered slopes in hemiarctic *Larix* and crooked forests (mainly *Pinus pumila* stands), hummocks in oligotrophic swamps. Hemiarctic *Larix* forests, crooked forests, and mountain tundras. Recorded for **G-Omol**, **KOLY, O-Kol** and **OKHO** by Blagodatskikh and Duda [3].

***Ptilidium pulcherrimum*** (G.Web.) Vain.—per., spor.—170–840—**KOLY** (1), **O-Kol** (6), **OKHO** (7, 8). *Larix* and *Pinus pumila* bark, decaying wood, and rarely, crevices between stones of large-block rock field. Hemiarctic *Larix* forests, crooked forests belts. Recorded for **KOLY, O-Kol** and **OKHO** by Blagodatskikh and Duda [3].

***Radula complanata*** (L.) Dumort.—per., ant., gemm.—900–1400—**O-Kol** (5, 6), **OKHO** (7). More or less dry rocks and crevices in them. Mountain tundra belt.

***Radula obtusiloba*** Steph.—1150—**O-Kol** (6). Cliff crevice. Mountain tundra belt.

***Radula prolifera*** Arnell—per. –1150–1600—**O-Kol** (5, 6). Among mosses in the cover of moist mossy and dwarf shrub-moss tundras on the slopes, the edges of solifluction spots, and cliff crevices. Mountain tundra belt.

***Riccardia chamaedryfolia*** (With.) Grolle—ant., arch., spor.—40–1110—**KOLY** (3, 4), **O-Kol** (5), **OKHO** (8). Hollows between ridges in oligotrophic swamps, and less often in moist mossy swampy tundras. Hemiarctic *Larix* forests, mountain tundras.

***Riccardia latifrons*** (Lindb.) Lindb.—ant., arch., per.—10–1110—**KOLY** (1, 3, 4), **O-Kol** (5), **OKHO** (8). Ridges and hollows in oligotrophic *Sphagnum* bogs, moist rocks near watercourses in *Alnus* thickets, and side walls of hummocks in moist mossy tundras. Hemiarctic *Larix* forests, crooked forests, and mountain tundras

***Riccardia palmata*** (Hedw.) Carruth.—per., arch., spor.—800–1090—**KOLY** (4). Over *Sphagnum* hummocks in a swampy moss tundra. Mountain tundra belt. Recorded for **O-Kol** by Blagodatskikh and Duda [3].

***Riccia frostii*** Austin—spor.—180—**KOLY** (2). Loamy soil along the bank of the Kolyma River. Hemiarctic *Larix* forest belt.

***Riccia sorocarpa*** Bisch.—spor.—1150–1600—**O-Kol** (6). The central part of fine soil spots of cryogenic origin in moss and dwarf shrub-moss tundras. Mountain tundra belt.

***Ricciocarpos natans*** (L.) Corda—**G-Omol**, **KOLY** The species was not found in the collections we gathered, although found in the herbarium material collected in (1) Omsukchansky District, M.G. Khoreva 03.VII.2004 sine n. (VBGI) and (2) Tenkinsky District, ca. 7 km from Elochka Settlement, 2nd terrace of the Kolyma River, *Equisetum* swampy communities, 01.VIII.2003, N.V. Sinelnikova #155 (VBGI; KPABG)

***Saccobasis polita*** (Nees) Buch—gemm.—890—**O-Kol** (5), **OKHO** (7). Peaty outcrops along the lake shore, moist rocks near a stream. Hemiarctic *Larix* forests, mountain tundra.

***Sauteria alpina*** (Nees) Nees—ant., arch., spor.—650–1600—**KOLY** (1), **O-Kol** (6), **OKHO** (7). Peaty and fine soil banks of streams, moist fine soil spots of cryogenic origin in the tundra, and cliff crevices. Mountain tundra belt, rarely—crooked forests.

***Scapania apiculata*** Spruce—ant.—**O-Kol** (5). The base of a *Larix* trunk. Hemiarctic *Larix* forests. Recorded for **O-Kol** by Blagodatskikh and Duda [3].

***Scapania brevicaulis*** Tayl.—per., spor., gemm.—920–1400—**KOLY** (1), **O-Kol** (5, 6). The banks of sluggishly flowing streams in mossy tundras, moist crevices between stones in rock fields, the edges of fine soil spots of cryogenic origin, and less often, and cliffs along the stream. Mountain tundra belt.

***Scapania crassiretis*** Bryhn—gemm.—1150–1400—**O-Kol** (6). Crevices between stones in rocky fields and crevices in moist cliffs. Mountain tundra belt. Recorded for **KOLY** by Blagodatskikh and Duda [3].

***Scapania curta*** (Mart.) Dumort.—per., gemm.—5–1460—**KOLY** (1), **O-Kol** (6), **OKHO** (8). Moist fine soil along roadsides, cliffs along streams, edges of fine soil spots of cryogenic origin. Coastal cliffs, crooked forests, and mountain tundra belts. Recorded for **KOLY** by Blagodatskikh and Duda [3].

***Scapania cuspiduligera*** (Nees) Mull.Frib.—gemm.—920–1600—**KOLY** (1), **O-Kol** (5. 6). Peaty and rocky banks of streams in moist mossy tundras, cliffs near watercourses, edges of fine soil spots of cryogenic origin, and less often hollows in moist tundras. Mountain tundras, and less often crooked forests.

***Scapania degenii*** (Schiffn) Mull.Frib.—gemm.—510–1600—**KOLY** (1, 4), **O-Kol** (5, 6). Basides listed, the species was found in the herbarium collection from Omolon River, V.B. Dokuchayeva 11.VII.1979 (VBGI). Moist hollows, less often hummocks in wet mossy tundras on the products of destruction of limestones, basalts and serpentinites, banks of streams, cliffs near watercourses, and edges of fine soil spots of cryogenic origin. Crooked forests, mountain tundras. Recorded for **KOLY** by Blagodatskikh and Duda [3].

***Scapania gymnostomophila*** Kaal.—gemm.—580–2040—**KOLY** (1, 2), **O-Kol** (5, 6), **OKHO** (7, 8). Mossy banks of sluggishly flowing ponds, hollows in the tundra on the products of destruction of basalts, serpentinites and limestones, moist cliff crevices (mainly those of alkaline composition), and less often humus on wet steep slopes and fine soil in niches under the roots in sparse *Larix* forests on the slopes. Hemiarctic *Larix* forests, crooked forests, and mountain tundras. Figure 2F.

***Scapania hyperborea*** Joerg.—870–1580—**O-Kol** (5, 6). Hollows between hummocks in moist mossy tundras, mossy banks of sluggishly flowing ponds, moist edges of fine soil spots of cryogenic origin, and rarely moist cliff crevices. Mountain tundras, alpine wastelands, and rarely crooked forests. Recorded for **KOLY** by Blagodatskikh and Duda [3].

***Scapania irrigua*** (Nees) Nees—ant., per., gemm.—5–1600—**KOLY** (1, 2, 3, 4), **O-Kol** (5, 6), **OKHO** (7, 8). Fine soil, humus, stones, and rocks along watercourses (including temporary ones) and waterfalls, moist edges of fine soil spots of cryogenic origin, moist hollows in mossy tundras, moist clayey roadsides, crevices in seaside rocks, fine soil in snowbed habitats. Coastal rocks, hemiarctic *Larix* forests, crooked forests, and mountain tundra belts. Recorded for **KOLY** and **O-Kol** by Blagodatskikh and Duda [3].

***Scapania kaurinii*** Ryan—ant., arch., per., spor.—510–1600—**KOLY** (1, 4), **O-Kol** (6), **OKHO** (7). Crevices in rocks along streams, crevices between stones in rock fields, stones along streams, moss-covered edges of fine soil spots of cryogenic origin, and less often hollows in wet mossy tundras. Mountain tundra belt.

***Scapania ligulifolia*** (R.M. Schust.) R.M. Schust.—gemm.—1166—**O-Kol** (6). Hollows in the swampy *Sphagnum* tundra. Mountain tundra belt.

***Scapania* *lingulata*** H.Buch—ant., per., gemm., spor.—10–1000—**KOLY** (3), **O-Kol** (6), **OKHO** (8). Peaty and sandy-loamy banks of streams, clayey roadsides, and less often fine soil in crevices between stones in rock fields. Hemiarctic *Larix* forests, crooked forests, and mountain tundra belts.

***Scapania magadanica*** S.-S. Choi, Bakalin and B.Y. Sun—per., ant.—170–510—**OKHO** (7, 8). Crevices between stones in rock fields and cliff crevices. Crooked forests belt.

***Scapania microdonta*** (Mitt.) Müll.Frib.—gemm.—10–1960—**KOLY** (1, 4), **O-Kol** (5, 6), **OKHO** (7, 8). More or less dry crevices in rocky outcrops and gravelly barrens, crevices in dry to moderately moist cliffs, and rarely among mosses in dry dwarf shrub-moss-lichen tundras. Coastal cliffs, mountain tundras, and alpine wastelands. Recorded for **KOLY** and **OKHO** by Blagodatskikh and Duda [3] as *Macrodiplophyllum microdontum* (Mitt.) Perss.

***Scapania mucronata*** H. Buch—gemm.—10–1200—**KOLY** (3), **O-Kol** (6), **OKHO** (8). Moist cliffs and their crevices, peaty soil on the trail side. Coastal rocks, crooked forests, and mountain tundra belts. Recorded for **KOLY** and **OKHO** by Blagodatskikh and Duda [3].

***Scapania obcordata*** (Berggr.) S. W. Arnell—per., ant., spor.—320–1600—**KOLY** (1, 4), **O-Kol** (6), **OKHO** (8). Moist hollows between hummocks in an oligotrophic bog and in moist mossy tundras, the edges of moist fine soil spots of cryogenic origin, peaty banks of streams, and rarely clayey roadsides. Mountain tundra, less often crooked forest belts.

***Scapania obscura*** (Arnell et C.E.O. Jensen) Schiffn.—450—**OKHO** (8). Moist stones along a stream in a crooked forest. Crooked forest belt. Recorded for **KOLY** by Blagodatskikh and Duda [3].

***Scapania paludicola*** Loeske and Mull.Frib.—per., spor., gemm.—10–1180—**KOLY** (1, 3, 4), **O-Kol** (5, 6), **OKHO** (8). In addition to those listed, the species was found in the herbarium collection from Omolon River, [collector unknown] 21.VII.1979 24 (VBGI). Hollows between hummocks in moist tundras and oligotrophic swamps, moist slopes in *Larix* forests with *Sphagnum* cover, less often—roadsides, humus banks of streams. Hemiarctic *Larix* forests, crooked forests, and mountain tundra belts. Recorded for **KOLY** and **O-Kol** by Blagodatskikh and Duda [3].

***Scapania parvifolia*** Warnst.—per., ant., gemm. var. ***parvifolia***—10–1400—**KOLY** (1, 3, 4), **O-Kol** (5, 6), **OKHO** (7, 8). Peaty banks and slopes to watercourses, the sides of paths and roads, stones along the banks of ponds and streams, the edges of fine soil spots of cryogenic origin, and rarely moist decaying wood along streams. Hemiarctic *Larix* forests, crooked forests, and mountain tundra belts. Recorded for **KOLY** and **OKHO** by Blagodatskikh and Duda [3].

***Scapania parvifolia*** Warnst. var. ***grandiretis*** Schljak.—per.—650—**KOLY** (4). Moist humus along the bank of a stream. Hemiarctic *Larix* forest.

***Scapania preatervisa*** Meylan—gemm.—20–1580—**KOLY** (2), **O-Kol** (6), **OKHO** (8). More or less dry cliff crevices, less often the edges of fine soil spots of cryogenic origin. Coastal cliffs, mountain tundras, and alpine wastelands.

***Scapania rufidula*** Warnst.—gemm.—10—**OKHO** (8). **O-Kol** The last report is based on herbarium collection from Tenkinsky District, N. Sinelnikova 10.VIII.2007 37y (VBGI). Stones along the bank of the stream. Hemiarctic *Larix* forest.

***Scapania scandica*** (H.Arnell and Buch) Macv.—300—**KOLY** (2). Decaying wood on the bank of a stream in *Alnus* thickets. Crooked forest.

***Scapania simmonsii*** Bryhn et Kaal.—580–1600—**KOLY** (2), **O-Kol** (5, 6). **GO**. The report for the last district is based on the herbarium collection from Omsukchansky District, M.G. Khoreva 03.VII.2004 sine n. (VBGI). Moist tundras developed over the products of the destruction of limestones and basalts, moist banks of streams, and crevices in alkaline rocks. Mountain tundra belt. Figure 2G.

***Scapania sphaerifera*** H. Buch.—gemm.—300–1310—**KOLY** (1, 4), **O-Kol** (6), **OKHO** (7, 8). Crevices between stones in rock fields, less often cliff crevices in rocks (strictly acidic composition). Crooked forests, mountain tundra belts.

***Scapania spitsbergensis*** (Lindb.) Müll. Frib.—per., ant.—500–1960—**KOLY** (1, 3, 4), **O-Kol** (6). Wet crevices in cliffs (including those near watercourses), crevices between stones in rocky fields, and rarely hollows and side walls of hummocks in swampy tundras and crumbling soil slopes to streams. Mountain tundra belt. Figure 2H.

***Scapania subalpina*** (Nees ex Lindenb.) Dumort.—ant., per., spor., gemm.—10–1600—**KOLY** (1, 4), **O-Kol** (6), **OKHO** (7, 8). Rocks, stones and fine soil near watercourses and waterfalls, moist to wet slopes to streams and rivers, less often—peaty and loamy roadsides, edges of moist fine soil spots of cryogenic origin. Hemiarctic *Larix* forests, crooked forests, and mountain tundra belts. Recorded for **KOLY** and **OKHO** by Blagodatskikh and Duda [3].

***Scapania tundrae*** (Arnell) H. Buch—1100–1250—**KOLY** (1). Hollows between hummocks in *Sphagnum* and dwarf shrub-moss swampy tundras, and rarely in wet loamy roadsides. Mountain tundra belt.

***Scapania uliginosa*** (Lindenb.) Dumort.—Recorded for **KOLY** and **OKHO** by Blagodatskikh and Duda [3]. The cited reference provides citation for this taxon as “=*S. paludosa*” that makes unclear to which taxon in the current understanding this report belongs since the occurrence of the both taxa is possible in Magadan Province.

***Scapania undulata*** (L.) Dumort.—290–520—**KOLY** (3), **O-Kol** (6), **OKHO** (7, 8). Moss-covered or rocky banks of streams, vertical cliffs near waterfalls (commonly in the splash zone). Crooked forests belt. Recorded for **KOLY** and **O-Kol** by Blagodatskikh and Duda [3].

***Schistochilopsis incisa*** (Schrad.) Konst. (including *S. opacifolia* (Culm. ex Meyl.) Konst. following to Bakalin et al0])—per., spor., gemm.—10–1600—**KOLY** (1, 3, 4), **O-Kol** (5, 6), **OKHO** (7, 8). Peaty, rocky and sandy banks of streams (including stream bed in drying up streams), moist moss cushions and humus along the shores of lakes, moist crevices between stones in rock field and cliff crevices, the side walls of hummocks in moss tundras and oligotrophic swamps, moist cliffs near waterfalls. Coastal rocks, hemiarctic *Larix* forests, crooked forests, and mountain tundra belts. Recorded for **KOLY, O-Kol** and **OKHO** by Blagodatskikh and Duda [3] as *Lophozia incisa* (Schrad.) Dumort.

***Schljakovia kunzeana*** (Huebener) Konstant. et Vilnet—per., ant., gemm.—10–1600—**KOLY** (1, 3, 4), **O-Kol** (5, 6), **OKHO** (8). Hollows and side walls of hummocks in moist moss hummocky tundras and moss and moss-sedge bogs, peaty banks of sluggishly flowing streams and lakes, less often—moist crevices between stones of large-block rock field, the edges of fine soil spots of cryogenic origin, moist cliff crevices along watercourses. Hemiarctic *Larix* forests, crooked forests, and mountain tundra belts. Recorded for **KOLY** and **O-Kol** by Blagodatskikh and Duda [3] as *Barbilophozia kunzeana* (Huebener) Müll. Frib.

***Schljakovianthus quadrilobus*** (Lindb.) Konstant. et Vilnet var. ***quadrilobus***—per., spor.—10–1400—**KOLY** (2), **O-Kol** (5, 6), **OKHO** (8). Moist soil along the edges of fine soil spots of cryogenic origin, moist hollows in mossy tundras, the banks of streams, and less commonly, Moist cliff crevices and moss cushions along the lake shores. Hemiarctic *Larix* forests, crooked forests, and mountain tundra belts.

***Schljakovianthus quadrilobus*** var. ***glareosa*** (Jørg.) Konstant. and Vilnet—1000—**O-Kol** (6). Peaty bank of a stream in a crooked forest. Crooked forest belt.

***Solenostoma confertissimum*** (Nees) Schljak.—ant., arch., per.—20–1580—**KOLY** (1), **O-Kol** (5, 6), **OKHO** (8). Moist clay roadsides, edges of fine soil spots of cryogenic origin, moist rocks and fine soil along streams, and rarely moss-covered slopes to streams. Hemiarctic *Larix* forests, crooked forests, and mountain tundra belts.

***Solenostoma obscurum*** (A. Evans) R. M. Schust.—per., ant.—10–400—**OKHO** (7, 8). Moist cliffs and fine soil along streams. Crooked forests.

***Solenostoma pusillum*** (C. E. O. Jensen) Steph.—per., ant.—1100–1300—**KOLY** (1, 4), **OKHO** (8). Fine soil and clayey roadsides, edges of fine soil spots of cryogenic origin. Mountain tundra belt.

***Solenostoma rossicum*** Bakalin and Vilnet—ant., per., spor.—10–1600—**KOLY** (1, 4), **OKHO** (8). Moist fine soil along the edges of ponds, rocky crevices in seaside cliffs, fine soil spots of cryogenic origin in the tundra, roadsides. Coastal rocks, crooked forests, and mountain tundra belts.

***Solenostoma sphaerocarpum*** (Hook.) Steph.—ant., arch., per., spor. var. ***sphaerocarpum***—10–1250—**KOLY** (1, 4), **O-Kol** (6), **OKHO** (8). Fine soil in roadsides, fine soil spots of cryogenic origin, fine soil on slopes to streams. Hemiarctic *Larix* forests, crooked forests, and mountain tundra belts.

***Solenostoma sphaerocarpum*** (Hook.) Steph. var. ***nana*** (Nees) Schust.—per., ant.—1100–1250—**KOLY** (1). Moist roadsides. Mountain tundra belt.

***Solenostoma subellipticum*** (Lindb. ex Heeg) R.M. Schust.—per., ant., spor.—5–1600—**KOLY** (1, 4), **O-Kol** (5, 6), **OKHO** (7, 8). Moist rocks, humus and fine soil near streams, vertical cliffy walls in the splash zone near waterfalls, moist fine soil along roadsides, peaty shores of lakes (in the splash zone), moist turfs along the edges of fine soil spots of cryogenic origin. Hemiarctic *Larix* forests, crooked forests, and mountain tundra belts. Recorded for **OKHO** by Blagodatskikh and Duda [3] as *Jungermannia subelliptica* (Lindb. ex Heeg) Levier.

***Sphenolobus cavifolius*** (Buch and S.W. Arnell) Mull. Frib.—per.—880–1200—**KOLY** (1), **O-Kol** (5, 6). Hummocks and hollows between them in mossy tundras developed on the products of destruction of limestones, basalts and serpentinites. Mountain tundra, and rarely hemiarctic *Larix* forests belts.

***Sphenolobus minutus*** (Schreb.) Berggr.—ant., per., spor.—5–1600—**KOLY** (1, 2, 3, 4), **O-Kol** (5, 6), **OKHO** (7, 8).—Hummocks in mossy and dwarf shrub tundras and oligotrophic bogs, crevices in rocks, peaty banks of streams and lakes, edges of fine soil spots of cryogenic origin, niches under *Larix* roots. Sea coastal cliffs, hemiarctic *Larix* forests, crooked forests, and mountain tundra belts. Recorded for **KOLY** and **OKHO** by Blagodatskikh and Duda [3] as *Anastrophyllum minutum* (Schreb. ex Cranz) R.M. Schust.

***Sphenolobus saxicola*** (Schrad.) Steph.—per., spor.—450–1740—**KOLY** (1, 4), **O-Kol** (5, 6), **OKHO** (7). Crevices in rock fields and cliffy outliers on ridgelines, edges of dry fine soil spots of cryogenic origin, hummocks in dry moss-lichen, and other types of tundras. Mountain tundra, alpine heaths, and rarely crooked forests and hemiarctic *Larix* forests belts. Recorded for **KOLY**, **O-Kol** and **OKHO** by Blagodatskikh and Duda [3] as *Anastrophyllum saxicola* (Schrad.) R. M. Schust.

***Tetralophozia setiformis*** (Ehrh.) Schljak.—50–1740—**KOLY** (1, 4), **O-Kol** (5, 6), **OKHO** (7, 8). More or less dry crevices, less often open surfaces of stones in rock fields, crevices in open and fairly dry cliffs, edges of dry fine soil spots of cryogenic origin, mossy cushions in alpine heaths and gravelly and moss-lichen tundras. Mountain tundras and alpine wastelands, and rarely crooked forests belts. Recorded for **KOLY, O-Kol,** and **OKHO** by Blagodatskikh and Duda [3].

***Trilophozia quinquedentata*** (Huds.) Bakalin—per., gemm.—10–1600—**KOLY** (1, 2, 3, 4), **O-Kol** (5, 6), **OKHO** (7, 8). Stones, fine soil and humus along the banks of watercourses (including temporary ones), cliffs along streams and near waterfalls, moist crevices in rocks, moist moss cushions in tundras of various compositions, crooked forests, and hemiarctic *Larix* forests, moist peaty and mossy slopes, the edges of fine soil spots of cryogenic origin. Sea coastal rocks, hemiarctic *Larix* forests, crooked forests, and mountain tundra belts. Recorded for **KOLY** and **OKHO** by Blagodatskikh and Duda [3] as *Tritomaria quinquedentata* (Huds.) H. Buch.

***Tritomaria exsecta*** (Schmid. ex Schrad.) Loeske—gemm.—1260—**O**-**Kol** (6). The side wall of a hummock in the mossy tundra on a slope. Mountain tundra belt.

***Tritomaria exsectiformis*** (Breidl.) Schiffn. ex Loeske—gemm.—10–1400—**KOLY** (1, 2), **O-Kol** (6), **OKHO** (7, 8). Mossy hummocks and the edges of fine soil spots in dwarf shrub-moss and mossy tundras, decaying wood in *Larix* forests, humus slopes to streams, crevices in granite cliffs. Hemiarctic *Larix* forests, crooked forests, and mountain tundra belts.

***Tritomaria scitula*** (Tayl.) Joerg.—gemm.—650–1400—**KOLY** (1), **O-Kol** (5, 6). Peaty and fine soil banks of streams, moist crevices in alkaline rocks. Crooked forests, mountain tundras, and rarely hemiarctic *Larix* forest belts. Recorded for **KOLY** by Blagodatskikh and Duda [3].

#### Doubtful Records (Not Included in Analysis)

*Calypogeia azurea* Stotler and Crotz—recorded for **KOLY** by Blagodatskikh and Duda [3]. This species does not occur in the Russian Far East in a recent revision by Buczkovska et al. [15], being replaced by the newly described *C. orientalis* Buczk. et Bakalin. However, the probability of occurrence of *C. orientalis* in Magadan Province is very low. Moreover, since to the best of our knowledge, all material collected by Blagodatskikh was identified by Duda after drying, the information on oil bodies (the only reliable character for definitively differentiating the taxon from morphologically similar taxa, such as *C. muelleriana*) was not available to Duda.

*Fuscocephaloziopsis connivens* (Dicks.) Váňa and L. Söderstr.—recorded for **KOLY** by Blagodatskikh and Duda [3] as *Cephalozia connivens* (Dicks.) Lindb., but the probability of occurrence of this taxon in Magadan Province is very low. The specimen most likely belongs to the lax modification of *Fuscocephaloziopsis pleniceps*.

*Marsupella tubulosa* Steph.—recorded for **KOLY** by Blagodatskikh and Duda [3] as *Marsupella emarginata* ssp. *tubulosa* (Steph.) N. Kitag. However, the identification feature widely used at that time to differentiate it from *M. emarginata* s. str. (unequal of leaf lobes) in the vast majority of cases is not applicable. The only reliable feature is biconcentric oil bodies (cf. [16]), a feature neglected at that time and not available in previously-dried material. *Marsupella tubulosa* has a boreal to temperate amphi-Pacific East Asian distribution, with the northernmost confirmed records from East Kamchatka, and the probability of occurrence of this taxon in Magadan Province is very low.

*Scapania nemorea* (L.) Grolle—recorded for **KOLY** by Blagodatskikh and Duda [3]. However, this taxon does not occur in the Russian Far East [17], and the report most likely belongs to *S. crassiretis*.

*Scapania verrucosa* Heeg—recorded for **KOLY, O-Kol** and **OKHO** by Blagodatskikh and Duda [3]. However, this taxon is not known in the northern part of the Russian Far East (although it is known as a rarity in its southern part). The cited report most likely belongs to *S. sphaerifera*, a species poorly understood at the time when treatment by Blagodatskikh and Duda [3] was conducted.

### 2.3. Comparison of the Regional Floras in Northeast Asia

**Figure 3:**
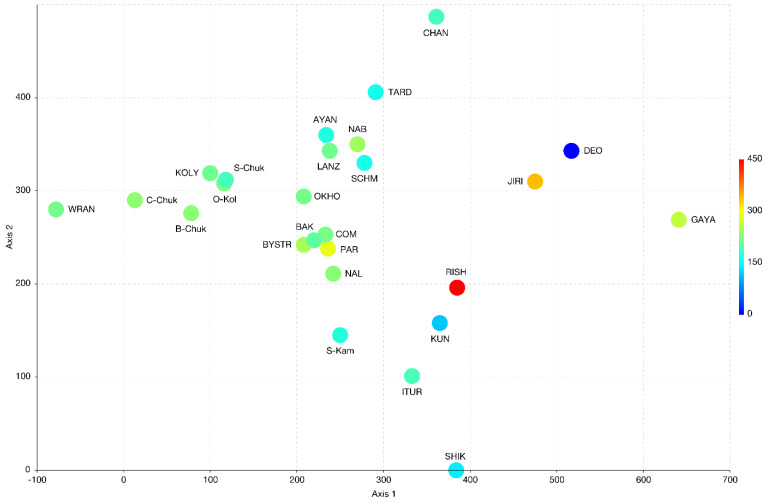
Comparison of the regional flora distribution in the DCA bubble chart (the third axis is the color gradient from deep blue to deep red).

**Figure 4:**
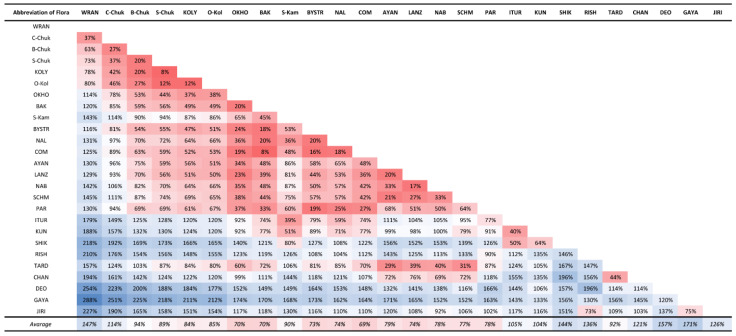
Distances between floras in Pacific Asia. The degree of proximity of floras, in comparison with the average distance, is highlighted by a color scheme from bright pink for the closest (starting from 8% of the total distance), through intermediate (white) to the most distant (blue, with a maximum of 288% of the total distance).

The results of the DCA analysis are visualized in Figure 3 and Figure 4. Clustering based on conditional distances (compared to the conditional average, as described in Materials and Methods) is given below. The provided list shows that with the requirement of “three neighbors”, clusters are allocated at a threshold value from 20 to 100% (of the length of the matrix average), while two clusters are allocated only at threshold values of 20 and 30%. For larger values, there is only one cluster, which includes more and more floras as the maximum permissible conditional distance increases. With the minimum requirement of “two neighbors”, 2 clusters are allocated at 10, 30, 80, and 90%. At 20% of the mean distance, three clusters are identified, and in other cases (40–70%), only one cluster is identified.

#### List of Clusters

10% of mean value distance between compared floras, three neighbors.

No clusters

20% of mean value distance between compared floras, 3 neighbors
Cluster 1: S-Chuk, KOLY, O-Kol
Cluster 2: BAK, BYSTR, COM

30% of mean value distance between compared floras, 3 neighbors
Cluster 1: OKHO, BAK, BYSTR, NAL, COM, AYAN, LANZ, SCHM, PAR
Cluster 2: B-Chuk, S-Chuk, KOLY, O-Kol

40% of mean value distance between compared floras, 3 neighbors
Cluster 1: C-Chuk, B-Chuk, S-Chuk, KOLY, O-Kol, OKHO, BAK, BYSTR, NAL, COM, AYAN, LANZ, NAB, SCHM, PAR, TARD

50% of mean value distance between compared floras, 3 neighbors
Cluster 1: C-Chuk, B-Chuk, S-Chuk, KOLY, O-Kol, OKHO, BAK, S-Kam, BYSTR, NAL, COM, AYAN, LANZ, NAB, SCHM, PAR, ITUR, TARD

60% of mean value distance between compared floras, 3 neighbors
Cluster 1: C-Chuk, B-Chuk, S-Chuk, KOLY, O-Kol, OKHO, BAK, S-Kam, BYSTR, NAL, COM, AYAN, LANZ, NAB, SCHM, PAR, ITUR, KUN, TARD

70% of mean value distance between compared floras, 3 neighbors
Cluster 1: WRAN, C-Chuk, B-Chuk, S-Chuk, KOLY, O-Kol, OKHO, BAK, S-Kam, BYSTR, NAL, COM, AYAN, LANZ, NAB, SCHM, PAR, ITUR, KUN, SHIK, TARD

80% of mean value distance between compared floras, 3 neighbors
Cluster 1: WRAN, C-Chuk, B-Chuk, S-Chuk, KOLY, O-Kol, OKHO, BAK, S-Kam, BYSTR, NAL, COM, AYAN, LANZ, NAB, SCHM, PAR, ITUR, KUN, SHIK, TARD, CHAN

90% of mean value distance between compared floras, 3 neighbors
Cluster 1: WRAN, C-Chuk, B-Chuk, S-Chuk, KOLY, O-Kol, OKHO, BAK, S-Kam, BYSTR, NAL, COM, AYAN, LANZ, NAB, SCHM, PAR, ITUR, KUN, SHIK, TARD, CHAN

100% of mean value distance between compared floras, 3 neighbors
Cluster 1: WRAN, C-Chuk, B-Chuk, S-Chuk, KOLY, O-Kol, OKHO, BAK, S-Kam, BYSTR, NAL, COM, AYAN, LANZ, NAB, SCHM, PAR, ITUR, KUN, SHIK, RISH, TARD, CHAN, JIRI

10% of mean value distance between compared floras, 2 neighbors
Cluster 1: BAK, COM
Cluster 2: S-Chuk, KOLY

20% of mean value distance between compared floras, 2 neighbors
Cluster 1: LANZ, NAB
Cluster 2: S-Chuk, KOLY, O-Kol
Cluster 3: OKHO, BAK, BYSTR, NAL, COM, PAR

30% of mean value distance between compared floras, 2 neighbors
Cluster 1: C-Chuk, B-Chuk, S-Chuk, KOLY, O-Kol
Cluster 2: OKHO, BAK, BYSTR, NAL, COM, AYAN, LANZ, NAB, SCHM, PAR

40% of mean value distance between compared floras, 2 neighbors
Cluster 1: WRAN, C-Chuk, B-Chuk, S-Chuk, KOLY, O-Kol, OKHO, BAK, S-Kam, BYSTR, NAL, COM, AYAN, LANZ, NAB, SCHM, PAR, TARD

50% of mean value distance between compared floras, 2 neighbors
Cluster 1: WRAN, C-Chuk, B-Chuk, S-Chuk, KOLY, O-Kol, OKHO, BAK, S-Kam, BYSTR, NAL, COM, AYAN, LANZ, NAB, SCHM, PAR, ITUR, KUN, TARD, CHAN

60% of mean value distance between compared floras, 2 neighbors
Cluster 1: WRAN, C-Chuk, B-Chuk, S-Chuk, KOLY, O-Kol, OKHO, BAK, S-Kam, BYSTR, NAL, COM, AYAN, LANZ, NAB, SCHM, PAR, ITUR, KUN, SHIK, TARD, CHAN

70% of mean value distance between compared floras, 2 neighbors
Cluster 1: WRAN, C-Chuk, B-Chuk, S-Chuk, KOLY, O-Kol, OKHO, BAK, S-Kam, BYSTR, NAL, COM, AYAN, LANZ, NAB, SCHM, PAR, ITUR, KUN, SHIK, TARD, CHAN

80% of mean value distance between compared floras, 2 neighbors
Cluster 1: RISH, GAYA, JIRI
Cluster 2: WRAN, C-Chuk, B-Chuk, S-Chuk, KOLY, O-Kol, OKHO, BAK, S-Kam, BYSTR, NAL, COM, AYAN, LANZ, NAB, SCHM, PAR, ITUR, KUN, SHIK, TARD, CHAN

90% of mean value distance between compared floras, 2 neighbors
Cluster 1: RISH, GAYA, JIRI
Cluster 2: WRAN, C-Chuk, B-Chuk, S-Chuk, KOLY, O-Kol, OKHO, BAK, S-Kam, BYSTR, NAL, COM, AYAN, LANZ, NAB, SCHM, PAR, ITUR, KUN, SHIK, TARD, CHAN

100% of mean value distance between compared floras, 2 neighbors
Cluster 1: WRAN, C-Chuk, B-Chuk, S-Chuk, KOLY, O-Kol, OKHO, BAK, S-Kam, BYSTR, NAL, COM, AYAN, LANZ, NAB, SCHM, PAR, ITUR, KUN, SHIK, RISH, TARD, CHAN, GAYA, JIRI

## 3. Discussion

The present study revealed a fairly high taxonomic diversity of liverworts, surpassing that of Chukotka (177 taxa cf. [18]) and comparable to that of the Republic of Yakutia (215 taxa, including 196 species cf. [19]), despite a much smaller area (562 thousand square kilometers versus three million square kilometers in Yakutia). The diversity of liverworts is somewhat higher in Kamchatka and on adjacent islands (232 species cf. [20]). This difference is quite natural and is explained by a more humid climate and, presumably, by a higher diversity of communities. At the same time, it is obvious that the potential for new records in Magadan Province has not been exhausted. The territory of the province is still studied very unevenly: there are practically no data on the flora of the entire eastern half of the province, where there are types of communities and types of habitats not represented in its western part.

The high taxonomic diversity of liverworts in Magadan Province is explained by its geographical location and natural conditions. The first thing to note is its position within ‘Mega-Beringia’, as a part of a single area not covered by a continuous glacier and located near the Bering Land Bridge. This circumstance explains the presence of a large number of Beringian and Mega-Beringian taxa. For some of these Beringian taxa, Magadan Province is the largest current enclave of distribution. The second feature that determines the high diversity of the flora is the widespread occurrence of basic rocks (basalts, limestones), which makes the existence of a large basiphylous complex within the flora inevitable. The third important feature is the distribution of almost true boreal forests (with a complex of boreal species, including the epixylous fraction) in the narrow strip along the Sea of Okhotsk, with the dominance, in general, of typical hemiarctic communities at the lower altitudinal levels in most of the area. Moreover, in the upper part of the altitudinal profile, tundra and alpine wastelands are widely developed, which inevitably enrich the flora with arctic–montane species.

When dividing the floras included in the analysis for comparison with both “two neighbors” and “three neighbors”, with minimal distance (20–30% of the average conditional distance), two patterns are confirmed: (1) the close connection of the Kolymsko-Okhotsky and Kolymsky floristic districts with the floras of Chukotka and (2) the connection of the Okhotsky floristic district of Magadan Province with the hemiarctic suboceanic floras of Kamchatka. When the distance is increased to 40%, almost all boreal, hemiarctic, and even hemiboreal floras included in the analysis (up to the Changbai Mts. and the southern Kurils) are combined into one cluster. Southern warm-temperate (in the lower altitudinal level) floras (mountain systems of South Korea) merge into one cluster with hemiarctic floras only when the minimal distance reaches 100% of the average and this already reflects only the most general relationship of floras in Pacific Asia.

In light of our statements on the incompleteness of the study of the liverwort flora of Magadan Province, it would be appropriate to make a short comparison with the state of knowledge of mosses and vascular plants of this province. The latest checklist of mosses from the Magadan region was published by Pisarenko and Bakalin [4]. It includes 364 species of mosses (of which 133 are recorded for the province for the first time). Thus, the number of known species of mosses exceeds the number of known liverworts by less than twice. On the one hand, this may suggest that the state of knowledge of liverworts is higher than that of mosses, and on the other hand, it may suggest that the state of knowledge of mosses is even worse than that of liverworts, because data on liverworts are also incomplete. Data on the distribution of mosses suffer from the same organic incompleteness as data on liverworts: almost all species known in the province are from only the three westernmost districts of the six in the province. And in this sense, their potential diversity has not been fully revealed, as is the case with liverworts. Vascular plants have been studied much better in Magadan Province than mosses and liverworts. The latest summary of flora [5] lists 1441 species of vascular plants (1168 excluding aliens). At the same time, there is significant information on the distribution of vascular plants in the eastern part of the province, although there are still fewer collection points in it than in the western half of the region. The flora of vascular plants of the Magadan region is much richer than the flora of vascular plants of Kamchatka (whereas poorer in mosses and liverworts), which once again emphasizes the untapped potential of bryophyte research in the region.

In the adjacent and huge (six times larger than Magadan Province) Republic of Yakutia, 1965 species of vascular plants are known (that is, 30% more than in Magadan Province) [19]. The difference is due to the larger area and probably greater diversity of habitats. However, if one compares the numbers of known species of liverworts and mosses in the Republic of Yakutia, the comparison is not always in favor of the latter. In Yakutia, 196 liverwort species are known (versus 205 in Magadan Province), which can be explained both by incomplete study (a factor characteristic of both regions) and by the fact that taxa with amphi-Pacific area are absent or few in number in the liverwort flora of Yakutia. At the same time, 517 species of mosses are known in Yakutia (versus 364 in Magadan Province), which, in addition to being understudied, can be explained by the lower “importance” of humidity for mosses than for liverworts and the deep penetration of Central Asian species there. 

The statistical analysis, as presented in this work, concerns only the Asian part of Beringia and the adjacent areas in Asia. However, apparently, species with a similar range are also common in the Western Hemisphere, in Alaska. Since we have not presented a mathematical comparison of the floras of Magadan Province with Alaska, it seems appropriate to briefly discuss here the connections between the liverwort flora of Magadan and the liverwort flora of Alaska. The liverwort flora of Alaska (excluding the Aleutian Islands) houses 267 species, that is, much richer taxonomically, but also larger in area. In Alaska, as well as in Magadan Province, *Apotreubia nana* is disjunctively distributed. *Asterella lindenbergiana* is occasionally found. The poorly studied rare *Barbilophozia rubescens* is not yet known there, although it may well be found in Alaska. The flora of Alaska has a larger number of species of more southern distribution. For example, the genus *Bazzania* is represented there by five species, versus two in Magadan Province. Moreover, it should be noted that species known from Alaska, but not known in Magadan Province, can hardly be expected on the territory of the latter. *Crossocalyx hellerianus* and *Scapania apiculata*, known in Pacific Asia from the northernmost points in Magadan Province, are known in Pacific America from Alaska. The same must be said about the penetration of *Neoorthocaulis attenuatus*, *Tritomaria exsecta*, *T. exsectiformis,* etc. to the north. Rare, as in Magadan Province, is *Fuscocephaloziopsis loitlesbergeri*. *Harpanthus scutatus*, known from Magadan Province (although highly distanced from the area core and generally extremely unexpected here), is not known in Alaska. *Lophozia lantratoviae* is also unknown in Alaska. It is unlikely to be found there. This is a hemiboreal Asian species that does not penetrate America. While recently described *Lophozia fuscovirens*, a little-known taxon, can be found in Alaska as well, *Mylia verrucosa*, an East Asian temperate species, is also unknown in Alaska and unlikely to be expected. The montane Asian *Scapania rufidula* is also absent from Alaska. The East Asian *Radula obtusiloba* is replaced in Alaska by the vicarious *Radula polyclada* (differences between the two taxa have not been reliably tested). Beringian and conditionally Beringian (possessing, in general, a wider range, but whose core is located in Beringia) closely connect Alaska and Magadan Province. This group is represented by *Cryptocolea imbricata*, *Eocalypogeia schusteriana*, *Frullania subarctica*, *Herbertus arcticus*, *Lejeunea alaskana*, *Lophozia schusteriana*, *Marsupella arctica*, *Plagiochila arctica*, *Pseudolepicolea fryei*, *Radula prolifera*, *Scapania ligulifolia*. Probably, *Frullania ignatovii* should also be included there, if we assume that the information about *F. eboracensis* Lehm. from Alaska actually belong to *F. ignatovii*. *Scapania magadanica*, so far known only from the Magadan region and Kamchatka, is probably a Beringian species; however, it is quite possible that it is limited to the Asian part of Beringia. The flora of Alaska turns out to be richer, largely due to more moisture-dependent and presumable temperate taxa. It is unlikely that the following species known in Alaska will ever be found in Magadan Province: *Acrobolbus ciliatus* (Mitt.) Schiffn., *Anastrepta orcadensis* (Hook.) Schiffn., *Cololejeunea macounii* (Spruce) A. Evans, *Douinia imbricata* (M. Howe) Constant. et Vilnet, *Metzgeria leptoneura* Spruce, *Nardia compressa* (Hook.) Gray, *Plagiochila semidecurrens* (Lehm. et Lindenb.) Lindenb., *Pleurozia purpurea* Lindb., etc. At the same time, finding several species that are not yet known in Magadan Province, but are found in Alaska, is quite possible in Magadan Province. This applies to *Anastrophyllum assimile* (Mitt.) Steph., *Ascidiota blepharophylla* C. Massal., *Gymnocolea fascinifera* Potemkin, *Gymnomitrion mucrophorum* R.M. Schust., *Hygrobiella laxifolia* (Hook.) Spruce, etc. The latter comparison shows that still there are several taxa that can be found in Magadan Province, but, in any way, their number is much less than is known to be found in Alaska.

## 4. Materials and Methods

### 4.1. Study Area

Since the description of the study area was given in detail earlier in a paper devoted to Magadan mosses [4], only the most important features need to be mentioned here.

Historically, Magadan Province was never covered by shield glaciation. Glaciation, if present, had a network or mountain–valley character. In addition, it should also be taken into account that the southern tip of Magadan Province, now washed by the waters of the Sea of Okhotsk, was a purely continental region during the maritime regressions. The coastline was located 100 km or more to the south, neither Gizhiga Bay nor Penzhina Bay in the modern sense existed, and this vast space was united by land with the territory of the modern isthmus of the Kamchatka Peninsula. Thus, the climate was ultracontinental throughout the current borders of Magadan Province, supporting the existence of tundra–steppes, which have remained under slightly altered conditions to the present day [1,2].

A peculiar feature of Magadan Province is the wide distribution of neutral (shales are the most common) and alkaline (limestones, basalts) substrates, including various combinations of both types. Acidic rocks, such as granites, are distributed locally, which invariably leads to the relative rarity of some acidophilous taxa commonly widespread in the hemiarctic. Over most of the region, mid-mountain relief is developed. At the same time, the mountains have a smoothed appearance (‘Goletz’ type), with a flat top and gentle slopes. More or less extensive bedrock outcrops are found only sporadically. The highest localities of the province are the Snezhnaya (2293 m) and Aborigen (2286 m) mountains, although in general, there are numerous peaks either almost reaching or slightly exceeding 2000 m above sea level.

A striking feature of Magadan Province is its continental climate with minimal precipitation. In the south of Magadan Province, the amount of precipitation reaches 660 mm per year (half in the form of liquid precipitation, https://en.climate-data.org/asia/russian-federation/magadan-oblast/magadan-986641/ accessed on 15 August 2023), while in the interior lands of the province, the amount of precipitation drops to 417 mm per year (Seymchan, https://en.climate-data.org/asia/russian-federation/magadan-oblast/seymchan-52370/ accessed on 15 August 2023) and even 306 mm per year (Jack London Lake [21]). However, a particular lack of moisture is usually not observed due to the commonness of horizontal precipitation and low summer temperatures. The average annual temperature varies from −2 °C on the coast to −11 °C inland. Summer is short, and the frost-free period is either absent or reaches 100 days in coastal areas. Near the western border of Magadan Province is the cold pole of the Northern Hemisphere (Oymyakon Settlement); in the interior areas of the province, minimum temperatures often drop below −60 °C in January. The average temperature of the warmest month varies from +12 to +15 °C on the plains and in the valleys, falling with elevation and reaching only +7.9 °C on the slopes of Aborigen (Susuman district) at an altitude of 1650 m above sea level [20]. Meanwhile, on clear sunny days in mid-July, daytime temperatures in the interior areas of the province can rise to +30 °C and even higher. The entire region, except for the narrow coastal strip, is located in an area of continuous permafrost.

The zonal vegetation in the territory of Magadan Province is hemiarctic, mainly represented by sparse *Larix cajanderi* Mayr forests and thickets of *Pinus pumila* (Pall.) Regel. Moreover, *Pinus pumila* commonly does not form impassable thickets, but due to the low height of snow cover, it forms open communities growing in small clumps, usually less than 1–1.5 m in height. In the narrow coastal strip, however, well-developed forest communities are formed, sometimes with a dense undergrowth of *Pinus pumila*, and have a transitional character to the boreal type. In addition, it is necessary to mention small (mainly only in the riparian zone) communities of *Betula lanata* (Regel) V.N. Vassil. and floodplain forests in wide valleys, sometimes including *Populus* L. and *Chosenia* Nakai, but often limited to various species of *Salix* L. Above the crooked forests, either gravelly wastelands or various types of tundra are developed, of which the most common types are lichen and hummocky dwarf shrub–moss tundras. Peat accumulation in waterlogged communities in the region either does not occur or occurs slowly, so the distinctions between wet tundra and swamps are blurred. Floristic regionalization of Magadan Province was developed by Khokhrjakov [22] and confirmed by Berkutenko et al. [5]. The boundaries of the regions have a sublatitudinal extension and reflect a trend of increasing continentality rather than decreasing temperature.

### 4.2. Data

The data used to compile this checklist were literature data and our own collected specimens, as well as small collections loaned to us by our colleagues.

As mentioned above, the first and only list of liverworts published to date for Magadan Province is that by Blagodatskikh and Duda [3], and it includes 92 species. At the same time, *Scapania obcordata* is given by the authors only for the coast of Chaun Bay, on the coast of the East Siberian Sea, and not belonging to Magadan Province. Thus, the list contains information on the distribution of 91 species within the current borders of the province. The cited list is based on extremely limited literature data, as well as materials collected by Blagodatskikh and identified by Duda during the 15 years of previous work by Blagodatskikh in Magadan Province. It should be noted that Blagodatskikh was a bryologist in the narrow sense; that is, she worked mainly with mosses and collected liverworts along the way, which of course, affected the knowledge of the flora. Some of the specimens from the Blagodatskikh collection are in the herbarium of the LE (St. Petersburg), some are in the herbarium MAG (Magadan), duplicates of the collection are in the Duda Herbarium (Prague), and some are probably lost, so we did not check the questionable species reports. Most of the districts of Magadan Province are very poorly accessible, and therefore, the available data cover only half of the province.

**Figure 5:**
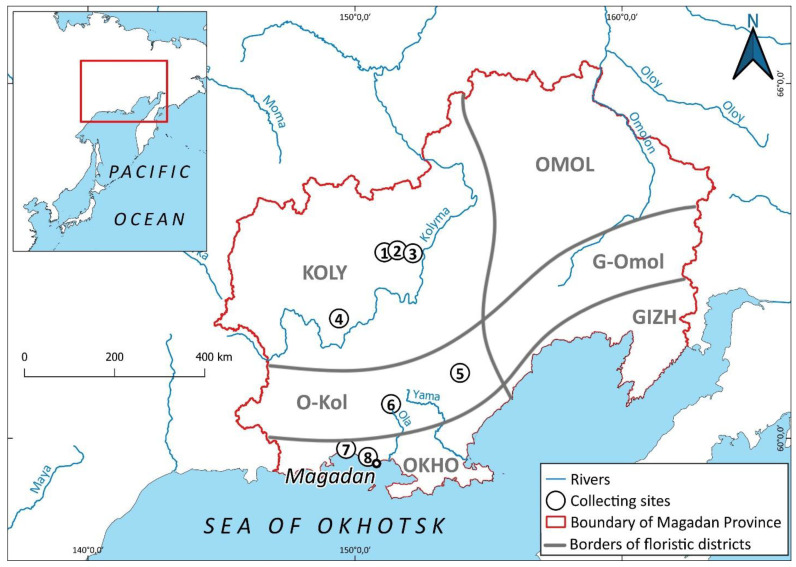
Collection sites (encircled numbers) in Magadan Province corresponding to Table 1. Abbreviations of floristic districts: KOLY—Kolymsky, O-Kol—Okhotstko-Kolymsky, OKHO—Okhotstky, OMOL—Omolonsky, G-Omol—Gizhiginsko-Omolonsky, GIZH—Gizhiginsky involved into analysis. The areas of OMOL, G-Omol, GIZH floristic districts were not studied. Floristic regionalization followed to Khokhrjakov [22] and Berkutenko et al. [5].

After the publication of the first checklist by Blagodatskikh and Duda [3], certain species were mentioned in taxonomic works on Russian Far East liverworts [17,23,24,25], etc. Our collections were carried out every year from 2010 to 2014, covering three floristic districts (out of 6). Data on the collection localities are presented in Table 1 and shown on the map (Figure 5). A total of approximately 3000 specimens were collected, approximately 20% of which were studied in the living state and thus for which information on oil bodies was available. For each collected specimen, geographic coordinates, elevation above sea level, vegetation type, substrate, moisture, and light conditions were recorded.

### 4.3. Phytogeographic Relationship Analysis

Floral relationship analysis was carried out using detrended correspondence analysis (DCA), which is applicable for datasets of various sizes and has been tested by us several times for amphi-Pacific liverwort floras [26,27,28,29,30]. The material for the analysis was our compiled matrix (Supplementary Table S1) of the species composition of floras, which is a summary species distribution of the compared floras, where the presence of a species is marked with the number 1, and its absence with the number 0. First, the coordinates in a three-dimensional grid were calculated for each of the compared floras (Supplementary Table S2), and then the distances between the floras were determined (Supplementary Table S3). The results were visualized in chart format, where the third axis is shown as a color gradient from red to blue. Given the features of the program, the abscissa is of primary importance for comparison, followed by the ordinate and, finally, the third axis, expressed in the diagram by a color gradient. Based on the distances between floras, the average value was calculated, and the relative distance between each of the floras included in the analysis was determined as a percentage of the average. Based on the relative lengths of the distances, clusters were identified, including floras, each of which is associated with at least **x** other floras at **y**% of the largest distance. **X** varied from 1 to 2, and **y**—from 10% to 100%. Therefore, the minimal number of clustered floras where **x** = 1 is two, while that where **x** = 2 is three.

The compared floras were selected based on two principles: territorial proximity to Magadan Province and revealed level (at least 75 species). In the matrix, we were forced to combine the floras compiled for floristic districts and well-studied ‘local’ floras. At the same time, regional liverwort floras of Magadan Province were included. This was done for the following reasons: (1) accessible and relatively complete information on the liverworts of Chukotka (i.e., the region with which comparison was especially important in the present work) is available for floristic districts only; (2) the studied localities in Magadan Province often do not fit the definition of ‘well-studied’ and to obtain more or less reliable information must be combined with nearby ones; (3) several floras included were not within Chukotka and Magadan Province, although they are ‘local’, in fact reflecting the flora of the entire region (for example, Southern Kamchatka, Central Kamchatka, etc.); and (4) floristic regionalization for the transition zone between the circumboreal region and the East Asian region is not carefully developed, especially in light of the fact thause the patterns of liverwort distribution differ from the patterns of distribution of vascular plants (cf. [31]). All floras involved in the analysis are described in Table 2.

## 5. Conclusions

The obtained data and the performed analysis show the high taxonomic diversity of the liverwort flora of Magadan Province and its tight connection with the floras of both Kamchatka and Chukotka. In fact, it is an integral part of a single (albeit regionally varying in detail) floristic complex of northeast Asia. The state of knowledge of the liverwort flora of Magadan Province is far from ideal: for half of the territory, there are practically no data. Therefore, it is advisable to conduct further research on liverworts in this area.

## Figures and Tables

**Table 1:** Liverwort collection sites.

N	Locality Description	Altitude Diapason	Latitude, N	Longitude, E	Collectors and Year
**Kolymsky Floristic District**					
1	Bolshoi Tuonnakh Mts.; Verina River basin	1100–2000 650–800	63°16′–63°18′ 63°15′–63°20′	151°02′–151°06′ 151°25′–151°32′	Bakalin and Choi, 2011
2	Upper course of Kolyma River, Zamok Cliff area	170–530	63°20′	152°35′	Bakalin and Choi, 2011
3	Basin of Seimchan River ca.60 km upstream of Seimchan Settl., Deryas’-Yurage River valley	350–700	63°17′	152°10′	Bakalin, 2010
4	Southern spurs of the Chersky Range, Bolshoi Anngachak Range	800–1600	62°07′–62°13′	149°18′–149°30′	Bakalin, 2014
**Okhotstko-Kolymsky floristic district**					
5	Watershed of Kilgana and Dzhugadzhaka Rivers	900–1400	61°11′–61°13′	153°54′–153°59′	Bakalin, 2012
6	Olskoye Basalt Plateau	850–1460	60°34′–60°42′	151°14′–151°32′	Bakalin, 2010; Bakalin and Choi, 2011; Bakalin, 2014
**Okhotstky floristic district**					
7	Coast of the Sea of Okhotsk, Kamennyi Range	400–1060	59°47′–59°49′	149°38′–149°42′	Bakalin, 2013
8	Coast of the Sea of Okhotsk, Oksa River basin; Magadan surroundings	0–100 0–700	59°39′ 59°35′	150°29′ 150°56′	Bakalin, 2010, 2012–2014; Bakalin and Choi, 2011

**Table 2:** Floras involved in the analysis.

No	Abbreviations of Floras	Explanation of the Abbreviation, Literature Sources	Approximate Coordinates
1	**AYAN**	Ayan Settlement surroundings, Dzhugdzhur Range, Pribrezhnyi Range [27]	56°27′ N 138°12′ E
2	**BAK**	Bakening Volcano and adjacent mountains in East Kamchatka [26]	54°00′ N 158°00′ E
3	**BYSTR**	Bystrinsky Nature Park, Sredinnyi Range, Central Kamchatka [32]	56°00′ N 158°30′ E
4	**B-Chuk**	Beringian Chukotka [18]	66°10′ N 175°25′ E
5	**CHAN**	Changbaishan Mts. in north-east China [33,34]	42°00′ N 128°00′ E
6	**C-Chuk**	Central Chukotka [18]	68°40′ N 173°53′ E
7	**COM**	Commander Islands [20,35]	55°00′ N 166°00′ E
8	**DEO**	Deokgyu Mts., Mt. Deogyu National Park, southern part of Korean Peninsula [36]	36°00′ N 127°30′ E
9	**GAYA**	Gayasan Mts., Gayasan Mountain National Park, southern part of Korean Peninsula [37]	35°48′ N 128°06′ E
10	**ITUR**	Iturup Island, Kuril Islands [26,38]	45°00′ N 149°00′ E
11	**JIRI**	Jirisan Mts, Jirisan National Park, southern part of Korean Peninsula [39]	35°20′ N 127°40′ E
12	**KOLY**	Kolymsky floristic district in Magadan Province (according to Figure 1) ([3], data from the present account)	63°20′ N 152°11′ E
13	**KUN**	Kunashir Island, Kuril Islands [26,28,38,40]	44°00′ N 146°00′ E
14	**LANZ**	Lanzhinskiye Mts. in North Okhotiya [41]	59°30′ N 143°30′ E
15	**NAB**	Nabilsky Range of Sakhalin [8]	51°00′ N 143°00′ E
16	**NAL**	Nalychevo Nature Park, Nalycheva River valley and adjacent volcanoes in East Kamchatka [42]	53°30′ N 159°00′ E
17	**OKHO**	Okhotstky floristic district in Magadan Province (according to Figure 1) ([3], data from the present account)	59°39′ N 150°29′ E
18	**O-Kol**	Okhotstko-Kolymsky floristic district in Magadan Province (according to Figure 1) ([3], data from the present account)	60°34′ N 151°14′ E
19	**PAR**	Paramushir Island, Kuril Islands [43]	51°30′ N 156°00′ E
20	**RISH**	Rishiri Island, opposite to western cost of Hokkaido Island [44]	45°00′ N 141°00′ E
21	**S-Chuk**	South Chukotka [18]	65°02′ N 170°20′ E
22	**SCHM**	Schmidt Peninsula in Sakhalin Island [8]	54°00′ N 142°30′ E
23	**SHIK**	Shikotan Island, Kuril Islands [26,28]	43°30′ N 143°30′ E
24	**S-Kam**	South Kamchatka Nature Park, South Kamchatka [45]	52°10′ N 158°00′ E
25	**TARD**	Tardoki Yani Range, northern part of Sikhote-Alin Mts [7]	48°53′ N 138°02′ E
26	**WRAN**	Wrangel Island [18]	71°14′ N 179°28′ E

## Data Availability

Data are contained within the article and Supplementary Materials.

## Supplementary Materials

The following supporting information can be downloaded at: https://www.mdpi.com/article/10.3390/plants12233928/s1, Table S1: Matrix of taxa distribution in compared floras for DCA analysis; Table S2: Normalized values of DCA for each compared flora; Table S3: Distances between the compared floras in arbitrary units based on the DCA analysis.
